# The Relative Roles of Selection and Drift in the Chaffinch Radiation (Aves: *Fringilla*) Across the Atlantic Archipelagos of Macaronesia

**DOI:** 10.1002/ece3.71307

**Published:** 2025-04-15

**Authors:** Brian Condori, María Recuerda, Juan Carlos Illera, Borja Milá

**Affiliations:** ^1^ Department of Biodiversity and Evolutionary Biology National Museum of Natural Sciences, Spanish National Research Council (CSIC) Madrid Spain; ^2^ Cornell Laboratory of Ornithology Cornell University Ithaca New York USA; ^3^ Biodiversity Research Institute (CSIC‐Oviedo University‐Principality of Asturias) University of Oviedo Mieres Spain

**Keywords:** adaptive and neutral divergence, *Fringilla*, genome–environment association analysis, islands, speciation

## Abstract

Island populations diverge from the mainland and from each other by both natural selection and neutral forces such as founder effects and genetic drift. In this work, we aim to determine the relative roles of selection and drift in the diversification of chaffinches (*Fringilla* spp.) in Macaronesia. We tested the hypothesis that taxa inhabiting Macaronesian archipelagos, which exhibit significant differences in habitat and climate compared with the mainland, should experience distinct ecological pressures, leading to divergence at loci under selection related to environmental variables. To determine the role of local adaptation in the differentiation of these taxa, we performed genotype–environment association (GEA) analyses using ten environmental variables and 52,306 single nucleotide polymorphism markers obtained from genotyping‐by‐sequencing (GBS) in 79 chaffinches. Redundancy analysis (RDA) revealed that genomic variation is associated with environmental variables and identified candidate genes related to phenotypic traits and environmental variables. Variables associated with habitat type and precipitation, together with drift, played an important role in the genomic differentiation between chaffinches from Macaronesia and the mainland, as well as within the Canarian archipelago. Genetic drift was identified as the main factor in the differentiation of North African chaffinches from Macaronesia and mainland Europe, as well as Madeira chaffinches from those in the Canary Islands. Finally, chaffinches from the Canary Islands show an incipient diversification process at the genetic and phenotypic level driven by both selection and neutral processes. Our results suggest that both habitat‐driven local adaptation and drift have played a role in the radiation of chaffinch taxa in Macaronesia.

## Graphical abstract

When colonizing oceanic archipelagos, island populations diverge through mechanisms of local adaptation and neutral processes. This study investigates the roles of selection and genetic drift in the radiation of chaffinches (*Fringilla* genus) in Macaronesia. Our findings reveal that habitat type and precipitation played an important role in the genomic differentiation between Macaronesian and European chaffinches, while African chaffinches have diverged mainly by drift.

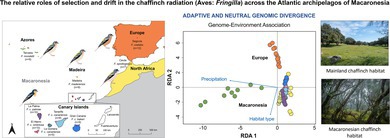

## 1 Introduction

Oceanic archipelagos are excellent systems for the study of divergence and speciation because of their geographic isolation, diversity of ecosystems, relatively small size, discrete borders, and reduced gene flow (Juan et al. 2000; Warren et al. 2015; Whittaker et al. 2017; Cumer et al. 2022; Illera et al. 2024). In these systems, speciation can occur through the divergence of colonizers from the continent via neutral and/or selective processes (Lamichhaney et al. 2015; Leroy et al. 2021), leading to an acceleration in diversification rates that can result in species radiations (De‐Kayne et al. 2024). However, distinguishing the role, intensity, and duration of each evolutionary force is challenging (Rundell and Price 2009; Grant and Grant 2024; Illera et al. 2024). In addition, adaptive and nonadaptive processes are not mutually exclusive and can operate concurrently (Seehausen et al. 2014; Gillespie et al. 2020; Illera et al. 2024). Adaptive radiations result in phenotypic and genotypic divergence among populations, as individuals maximize their fitness in different environments through local adaptation (Rundle and Nosil 2005; Grant and Grant 2008). Local adaptation results from a balance between selection and gene flow, where selection favors beneficial alleles in a given environment, while gene flow can either facilitate adaptation through adaptive introgression or counteract locally adapted alleles if it exceeds a critical threshold (Tigano and Friesen 2016). One of the most iconic examples of adaptive radiation in island birds includes the radiation of Darwin's finches (Thraupidae) in the Galápagos archipelago (Almén et al. 2016), in which the beaks of different species have evolved in response to the diverse trophic resources available. On the other hand, phenotypic and genotypic divergence in nonadaptive radiations can also arise through underlying mechanisms such as founder effects, bottlenecks, and genetic drift, as it can induce changes in signaling traits and lead to prezygotic reproductive isolation between populations (Panhuis et al. 2001; Rundell and Price 2009).

Although a single gene was found to be correlated with beak size in the radiation of Darwin's finches (Lamichhaney et al. 2016), most fitness traits are quantitative and polygenic, and it is likely that selection acts simultaneously on many loci of low effect, making their detection more challenging (Pritchard and Di Rienzo 2010). Establishing accurate genotype–phenotype maps based solely on genomic data presents significant challenges due to the inherent complexity of interactions among genes and between genes and their environments, factors that current analytical tools may not fully capture (Templeton 1998; Bazakos et al. 2017). Genotype–phenotype relationships can be highly intricate and are influenced by factors such as complex demographic histories and environmental traits which shape the genomic structure of populations. If not properly controlled for, this structure may lead to spurious associations where, for instance, observed links arise from demographic patterns rather than genuine trait associations (Simonin‐Wilmer et al. 2021). Furthermore, the exploration of interactions across different biological layers, such as the genome, epigenome, transcriptome, and metabolome, remains limited, posing an additional challenge to achieving a comprehensive understanding of complex phenotypes (Ritchie et al. 2015). Acknowledging these challenges highlights the importance of integrating complementary approaches to better elucidate the intricate nature of genotype–phenotype associations. However, advances in high‐throughput sequencing methods and the development of bioinformatic and statistical tools enable the detection of genomic footprints of recent adaptation and polygenic selection in order to find genetic evidence for adaptive divergence (Martin et al. 2024; Spurgin et al. 2024), given that high genomic coverage provides sufficient statistical power (Wang et al. 2015). Genotype–environment association methods [GEA; Hedrick et al. 1976] have proven to be effective in detecting loci linked to local adaptation (Forester et al. 2018), which could be drivers of population differentiation (e.g., Alvarado et al. 2022; Sheppard et al. 2024). GEA methods allow the detection of weak signals of polygenic selection distributed across the genome and can help to identify loci associated with environmental variation when using genome‐wide markers and relevant environmental variables (Hoban et al. 2016; Forester et al. 2018; Sheppard et al. 2022). In addition, GEA methods allow controlling for neutral population structure to avoid false positives, so that it becomes possible to assess the relative roles of drift and selection in shaping genetic variation across populations or recently diverged taxa (Bourgeois and Warren 2021).

The Common Chaffinch species complex in the genus *Fringilla* represents an optimal system for studying speciation on oceanic islands since their geographic range includes Eurasia, Northern Africa, and three north Atlantic oceanic archipelagos in Macaronesia: Azores, Madeira, and the Canary Islands (Shirihai and Svensson 2018). Previous studies have shown that the colonization of Macaronesian archipelagos occurred sequentially from the mainland, through the Azores and Madeira, to the Canary Islands, resulting in a species‐level radiation (Marshall and Baker 1999; Recuerda et al. 2021a). This radiation includes the Common Chaffinch ( *Fringilla coelebs* ) from Eurasia, and four recently recognized species corresponding to Northern Africa (*Fringilla spodiogenys*), Azores (*Fringilla moreletti*), Madeira (*Fringilla maderensis*), and the Canary Islands (*Fringilla canariensis*). While the chaffinches from Azores and Madeira are monotypic, four endemic subspecies have been recognized in the Canary Islands: *F. c. canariensis* from Tenerife and La Gomera, *F. c. palmae* from La Palma, *F. c. ombriosa* from El Hierro, and *F. c. bakeri* from Gran Canaria (Illera et al. 2018). The estimated origin of this colonization dates back to approximately 0.83 million years ago (Recuerda et al. 2021a). It occurred after the establishment of the Macaronesian subtropical laurel forests and moist heaths during the Plio‐Pleistocene (Kondraskov et al. 2015), habitats to which Macaronesian chaffinches are largely restricted. Macaronesian archipelagos differ in climate, with the Azores and Madeira having a higher average precipitation rate and lower mean temperatures compared with the Canary Islands (Cropper and Hanna 2014). Moreover, the Canary Islands exhibit a precipitation gradient where the western islands are wetter than the eastern ones (Sánchez‐Benítez et al. 2017). Dispersal in Macaronesian chaffinches appears to be limited across the islands. Previous studies suggest no evidence of dispersal movements among the different Canary Islands (Illera et al. 2018; Recuerda et al. 2021a) and that the Madeiran chaffinch only breeds on the island of Madeira (Rodrigues et al. 2014). In contrast, the lack of genetic structure among islands in the Azores indicates that they are connected by gene flow (Rodrigues et al. 2014). Additionally, Macaronesian chaffinches generally have shorter and more rounded wings, longer tarsi and bills, and larger bodies compared to continental populations (Grant 1979; Illera et al. 2018). Studies in both oscine and suboscine passerines suggest that dietary niche divergence exerts ecological selection on body size and beak shape, which in turn generates divergent vocal signals (Tobias et al. 2020). Thus, these phenotypic traits are likely to be under selection due to the distinct habitat types between island and mainland chaffinches, which could contribute to local adaptation in response to different ecological pressures.

Determining the relative roles of geographic isolation and local adaptation in driving divergence among populations on archipelagos and islands is crucial to understanding speciation processes in these systems. Here, we investigated the causes shaping the radiation of *Fringilla* chaffinches. Given the environmental variation between mainland and Macaronesian archipelagos, we predict that chaffinches under distinct ecological pressures should exhibit a higher proportion of genomic variance explained by loci under selection related to environmental variables. In turn, chaffinches under similar ecological pressures should exhibit divergence driven mostly by neutral factors (genetic drift). For example, we expect that genes related to beak shape and body size may be under selection in response to the different ecological niches of the island chaffinches compared to their mainland counterparts. To test this hypothesis, we performed GEA analyses to detect signatures of selection by examining the correlation between genomic variation and environmental variables, while controlling for neutral population structure. The specific objectives of this study were: (a) to assess the neutral genomic structure of the chaffinch species, (b) to detect adaptive genomic variation associated with differences in environmental traits among archipelagos and islands, and (c) to identify candidate genes under selection associated with environmental variables.

## 2 Methods

### 2.1 Study System and Sample Collection

**FIGURE 1:**
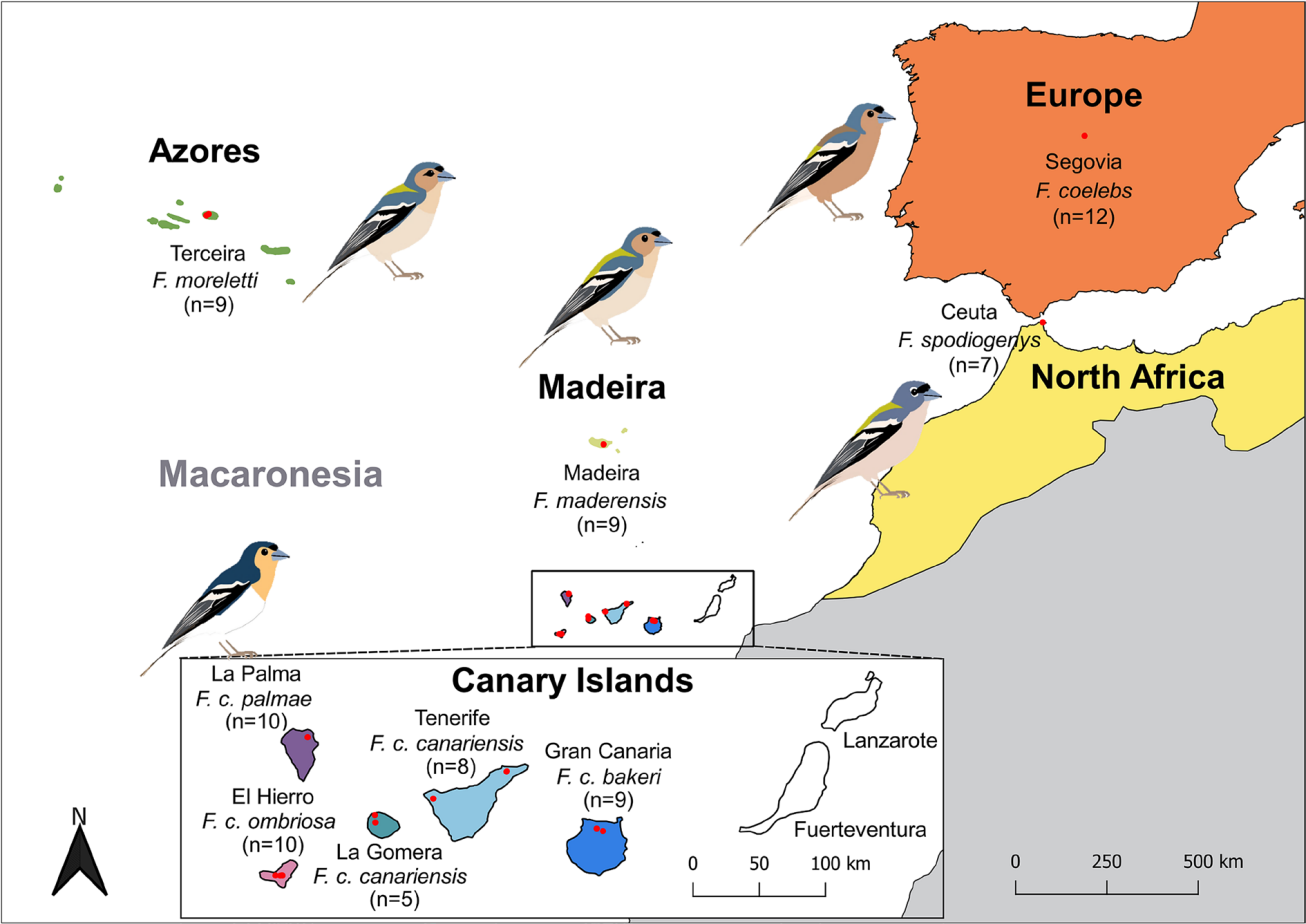
Distribution map of the chaffinch species analyzed in the present study. Note the species is absent in the eastern Canary Islands of Fuerteventura and Lanzarote. The red dots represent the sampling sites and the sample size is indicated in parentheses. Sketches depict the main phenotypic differences between species, with the Canary Islands chaffinches (*Fringilla canariensis*) represented by subspecies *F. c. palmae*.

For this study, genomic data were generated from blood samples of 79 adult males from wild populations in Europe (Segovia, central Spain), Northwest Africa (Ceuta), the Azores (Terceira Island), Madeira Island, and the five Canary Islands inhabited by the Canary Islands chaffinch (Gran Canaria, Tenerife, La Gomera, La Palma, and El Hierro). In total, the study included five species: *Fringilla coelebs* , *F. spodiogenys*, *F. moreletti*, *F. maderensis*; and all four subspecies of *F. canariensis* (Figure 1). The birds were captured using mist nets, and each individual was marked with a uniquely numbered aluminum ring to avoid resampling. Blood samples were obtained by venipuncture of the sub‐brachial vein and stored in absolute ethanol at −20°C in the laboratory before DNA extraction. Ringing activities were carried out with all the necessary permits from the Regional Governments from Azores, Madeira, Canary Islands, Ceuta, and Junta de Castilla y León. All experimental procedures were approved by the Commission of Bioethics at the University of Oviedo (PROAE 45/2019), and the CSIC Ethics Committee on Animal Experimentation (CEEA 1415/2023).

### 2.2 SNP Genotyping and Datasets

High‐quality genomic DNA was extracted from frozen blood preserved in ethanol using a QIAGEN Blood and Tissue kit (Qiagen, Valencia, CA) following the manufacturer's protocol. We used genotyping‐by‐sequencing (GBS) to obtain single nucleotide polymorphism (SNP) data (Elshire et al. 2011). This restriction site‐associated DNA sequencing technique uses genomic DNA digested with the enzyme *PstI* and posterior sequencing on an Illumina HiSeq X Ten platform. Raw reads were trimmed to remove low quality ends using TrimGalore v. 0.4.4 (Krueger 2015). Reads were mapped against the latest high‐quality version of the chromosome‐level chaffinch reference genome (GCA_015532645.2, Recuerda et al. 2021b) using the Burrows–Wheeler Aligner [BWA; Li and Durbin 2009] with the “‐mem” algorithm and default parameters. As mean depth coverage was 10.84 (Figure S1), variant calling was performed with GATK v. 3.6 HaplotypeCaller and GenotypeGVCFs tools (McKenna et al. 2010), calling all samples together with a minimum base and mapping quality score of 30. The variant dataset obtained was filtered using VCFtools v. 0.1.15 (Danecek et al. 2011) keeping biallelic loci with a coverage depth ranging between 5 and 60, a Phred quality score over 30, and a minor allele frequency (MAF) over 0.018. Indels were removed along with sites with over 75% missing data and showing significant deviation from Hardy–Weinberg equilibrium (*p* value < 10^−4^), resulting in a vcf file with 52,306 SNPs. To explore evolutionary processes at different spatial scales, we divided the dataset into multiple subsets. Using VCFtools, we created two more datasets: the second dataset with only the individuals from Madeira and the Canary Islands, and the third one with only the individuals from the Canaries (see Data S1 for details).

We used BayeScan v2.1 (Foll and Gaggiotti 2008) to separate neutral loci from loci under divergent selection. Briefly, this program detects selective loci identified as outlier SNPs in an *F*_ST_ distribution. We ran the program on the full dataset with the default sample size of 5000, a thinning interval of 200, a total of 20 pilot runs of 10,000 iterations each, and a burn‐in of 100,000 iterations. We checked for the convergence of MCMC chains and set the false discovery rate (FDR) parameter at 0.05. Subsequently, in order to assess population structure, we filtered the neutral dataset for linkage disequilibrium (LD) using PLINK v1.90 (Purcell et al. 2007; Chang et al. 2015) and set a *r* ^2^ threshold = 0.2, a window of 10 kb, and a window step size of 10, resulting in a final dataset of neutral unlinked SNPs from all populations. We also performed analyses to separate neutral loci from loci under selection on the dataset with populations from Madeira and the Canary Islands and on the dataset with the Canarian populations. Finally, we filtered the neutral dataset with the Canary Islands populations for LD to assess the Canarian population structure (see Data S1 for details).

### 2.3 Genome‐Wide Population Structure

To explore the genome‐wide population structure of the chaffinch radiation we performed a principal components analysis (PCA) using the neutral unlinked SNP dataset including all the populations and the neutral unlinked SNP dataset of the Canary Islands populations. We used LD‐pruned datasets to prevent a distorted view of population substructures and ensure data independence (Zou et al. 2010). The PCA was performed using the “glPca” function in the R package “adegenet” (Jombart 2008; Jombart and Ahmed 2011) in R v. 4.2.3 (R Core Team 2020). We decomposed the covariance matrix into eigenvectors, retaining the first two principal components (PCs) that explained the highest proportion of variance. The total number of eigenvectors was 78 (PCA with all populations) and 41 (PCA with Canarian populations), and the eigenvalues indicated the amount of variance explained by each principal component. These eigenvectors were used to explore population structure and visualize patterns of genetic variation across the samples. Additionally, to assess whether the main axes of genetic variation reflected patterns of isolation by distance (IBD), we computed geographic distances using the coordinates of each individual with the “distm” function from the R package “geosphere” (Hijmans 2024). We then calculated Euclidean distance matrices for both PC1 and PC2 using the “dist” function implemented in R. To formally test for IBD, we performed Mantel tests (Mantel 1967) between the geographic and Euclidean genetic distances, using the “mantel” function from the R package “vegan” (Oksanen et al. 2022), with 10,000 permutations to assess statistical significance.

### 2.4 Environmental Variables

To characterize the different habitats of the mainland and Macaronesia, we selected 10 remotely sensed environmental variables related to temperature, precipitation, and vegetation cover based on previous knowledge of the chaffinch ecology (Carrascal et al. 1992). The climatic variables were downloaded from WorldClim v. 2.1 (Fick and Hijmans 2017) and correspond to the 30 years average (1970–2000) of the following variables: annual mean temperature (Bio01), isothermality (Bio03), temperature seasonality (Bio04), mean temperature of the wettest quarter (Bio08), annual precipitation (Bio12), precipitation of the driest month (Bio14), precipitation seasonality (Bio15), and precipitation of the warmest quarter (Bio18). In addition, we downloaded two variables related to primary vegetal productivity: Normalized Difference Vegetation Index (NDVI) from the MODIS satellite from NASA, which is calculated every 16 days with a 250 m resolution since the year 2000 (Didan 2021); and tree cover data with a 10 m resolution for the year 2018 from the Copernicus satellite (https://land.copernicus.eu/).

### 2.5 Genotype–Environment Association Analysis

To assess the role of selection shaping genomic divergence across different populations, we performed a redundancy analysis (RDA; Legendre and Legendre 1998). RDA is an ordination approach which allows estimating the variance in a response variable (here, genomic variation), that can be explained by a set of explanatory variables (here, environmental variables). As a GEA method, RDA allows the detection of weak signals of polygenic selection across the genome and helps identify loci associated with environmental variables. We conducted this analysis using the complete dataset (including neutral SNPs, SNPs under selection, and SNPs without LD pruning) including all populations, the complete dataset with populations from Madeira and the Canary Islands, and the complete dataset only with populations from the Canary Islands. We performed the variable selection using the “forward.sel” function from the R package “adespatial” (Dray et al. 2022), which implements an algorithm to select the variables that best explain the spatial structure of the data. The selected variables aligned with our a priori expectations based on known ecological differences between the mainland and Macaronesia, as well as among Macaronesian archipelagos. As stated in the introduction, habitat type, precipitation, and temperature are key environmental factors distinguishing these regions, and these variables were also identified through our selection procedure. We evaluated the collinearity of the environmental variables ensuring that the selected variables had the lowest Pearson correlation value (|*r*| < 0.7), as recommended by Dormann et al. (2013). We ensured that the variance inflation factor (VIF) of the selected variables was below 10 and a permutation test was performed on the final RDA, as recommended by Borcard et al. (2018). The selected variables were: NDVI and precipitation of the driest month in the analyses with the dataset containing all populations; NDVI and annual mean temperature in the analyses of populations from Madeira and the Canary Islands; and NDVI and precipitation of the warmest quarter in the analyses of the Canarian dataset.

In addition, we performed a partial RDA (pRDA) which allows for the correction of the effects of a set of covariates. In this case, to control for the effect of genetic drift, the pRDA controlled for neutral population structure by including as a covariate the PC1 values from the genomic PCA performed with the datasets that only included neutral SNPs (Forester et al. 2018). Since these values reflected the pattern of isolation by distance, we aimed to avoid incorrect genotype–environment associations due to shared ancestry or geographic isolation (Cao et al. 2019; Gibson and Moyle 2020; Chang et al. 2022). As the RDA requires datasets without missing data, we imputed missing genotypes (17% in all datasets) using the most common genotype across all individuals in each dataset (Forester et al. 2018). To assess whether this imputation introduced significant bias, we also performed additional analyses using SNPs with less than 20% and 10% missing data prior to imputation. The function used for imputation is provided in the Data S1. The statistical significance of the complete and per‐axis models was tested using ANOVA‐like permutation tests setting *α* = 0.01 and using 1000 permutations. The analyses were conducted using the R package “vegan” (Oksanen et al. 2022). To evaluate whether incorporating additional PCs would improve control for neutral population structure, we repeated the pRDA analyses including PC1 through PC10 as covariates.

### 2.6 Candidate Loci Associated With Adaptive Divergence

We used RDA and pRDA analyses to estimate the correlation of outlier SNPs with the selected environmental variables as they are effective methods for detecting loci under selection even if they are of small effect (Forester et al. 2018; Capblancq and Forester 2021). We performed the outlier detection analyses by setting the threshold for considering outlier loci at ±3 standard deviations (SD) from the mean of the loading distribution of each axis. In the analyses of the Canary Islands dataset, we set the threshold for considering outlier loci at ±2.5 SD to increase the probability of detecting loci under weak selection in such a recent diversification (Forester et al. 2018). Finally, we identified the environmental variables that explained the most variance for each outlier SNP by correlating the observed allele frequencies across populations with each predictor (Forester et al. 2018). The absolute values of the loadings were represented in Manhattan plots using the R package “qqman” (Turner 2014). To identify candidate genes potentially associated with the observed adaptive divergence, we extracted the annotation of the SNPs putatively under selection detected for each variable from the common chaffinch genome annotation (gff file, Recuerda et al. 2021b) using bedtools intersect (Quinlan and Hall 2010). Finally, descriptions of putative functions and gene ontologies of the candidate genes were obtained through a bibliographic search at https://www.genecards.org/ (Stelzer et al. 2016).

## 3 Results

### 3.1 SNP Genotyping and Datasets

We generated three datasets corresponding to (i) all populations (52,306 SNPs), (ii) populations from Madeira and Canary Islands (19,909 SNPs), and (iii) populations from the Canary Islands (14,539 SNPs). In the dataset with all populations, we identified 51,463 neutral SNPs and 843 SNPs putatively under selection, of which 24 were under disruptive or directional selection as they presented high *F*_ST_ values, and 819 were under purifying selection, as they showed low *F*_ST_ values (Figure S2a). In the dataset with the populations from Madeira and the Canary Islands, we obtained 19,803 neutral SNPs and 106 SNPs putatively under selection, all of which were likely under purifying selection, as they exhibited low *F*_ST_ values (Figure S2b). In the dataset with the Canary Islands populations, we did not detect any candidate SNP loci under selection, so all loci in the dataset were identified as neutral. Finally, applying the LD filter, we obtained a total of 31,388 and 8100 neutral unlinked SNP loci in the dataset with all populations and in the dataset with the Canary Islands populations, respectively.

### 3.2 Genome‐Wide Population Structure in Neutral Loci

**FIGURE 2:**
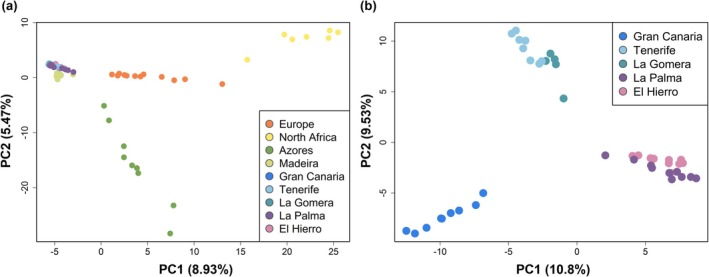
Genome‐wide population structure of chaffinches from mainland and Macaronesian populations: (a) PCA plot based on the first two principal components conducted on the dataset with all populations (31,388 neutral and independent SNPs) and (b) PCA based on the two principal components performed on the dataset with Canary Islands populations only (8100 neutral and independent SNPs).

The PCA of neutral unlinked loci showed marked structure between the five species, with Madeira and the Canary Islands forming a relatively tight cluster (Figure 2a). PC1 and PC2 explained 8.93% and 5.47% of the genomic variation, respectively. The North African populations showed the highest differentiation from the insular populations. Within the Canary Islands, the PCA showed a differentiated structure among the five populations from the different islands, although the Tenerife individuals clustered close to La Gomera, and those from La Palma close to El Hierro (Figure 2b). PC1 explained 10.8% of the genomic variation, and PC2 explained 9.53% (Figure 2b). The Mantel test revealed a significant correlation between geographic distance and genetic structure for both PC1 (*r* = 0.614, *p* = < 0.001) and PC2 (*r* = 0.695, *p* = < 0.001) when considering all populations. A similar pattern was observed in the dataset including only Madeira and the Canary Islands, where PC1 (*r* = 0.619, *p* = < 0.001) and PC2 (*r* = 0.331, < 0.001) showed significant correlations with geographic distance. In the Canary Islands dataset, PC1 remained significantly correlated with geographic distance (*r* = 0.744, *p* = < 0.001), whereas PC2 did not (*r* = 0.045, *p* = 0.165).

### 3.3 Adaptive Divergence Associated With Environmental Variables

#### 3.3.1 Macaronesia and Mainland Populations

**FIGURE 3:**
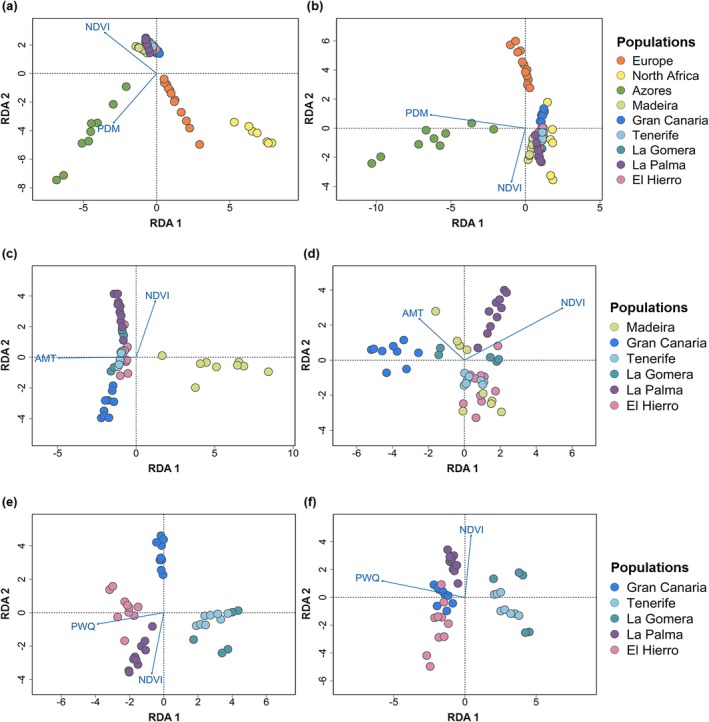
Adaptive and neutral divergence in the chaffinch radiation. Plots show results from redundancy analysis (RDA) performed with different datasets. (a) Regular RDA performed using the dataset with all populations. (b) Partial RDA (pRDA) controlling for neutral genetic structure performed using the full dataset. (c) Regular RDA performed using the dataset with populations from Madeira and the Canary Islands. (d) pRDA controlling for neutral genetic structure performed using the Madeira and Canary Islands dataset. (e) Regular RDA of Canary Islands populations. (f) pRDA of Canary Islands populations controlling for neutral genetic structure. Vectors indicate the environmental predictors: AMT, annual mean temperature; NDVI, Normalized difference vegetation index; PDM, precipitation of the driest month; PWQ, precipitation of the warmest quarter.

The RDA of the full dataset showed a clear habitat‐related structure, separating Macaronesian individuals (inhabiting humid cloud forests) and mainland individuals (inhabiting Mediterranean and temperate forests in Europe) along the first RDA axis, which was correlated with NDVI and explained 0.05% of the variation (Figure 3a). Macaronesian individuals were positively correlated with vegetation greenness (NDVI). On the other hand, Azorean individuals exhibited clear structure along the second RDA axis, which was positively associated with the precipitation of the driest month (PDM) and explained 0.04% of the variation (Figure 3a). Both the complete model and the first two axes were statistically significant. The pRDA analysis controlling for neutral structure also showed a separation between Macaronesian and European individuals along the second pRDA axis, which was correlated with NDVI and explained 0.02% of the variation (Figure 3b). In addition, the pRDA also revealed a clear structure of the Azorean individuals along the first pRDA axis, which was correlated with PDM and explained 0.05% of the variation (Figure 3b). In contrast, the cluster of individuals from North Africa moved toward the origin in the pRDA, reflecting a weak association with the environmental variables (Figure 3b). In these analyses, both the complete model and the first two axes were also statistically significant (*p* < 0.01). The analyses using SNPs with less than 20% and 10% missing data prior to imputation yielded results consistent with our original findings, suggesting that imputation did not introduce significant bias (Figure S3). Moreover, including additional PCs (PC1–PC10) as covariates in the pRDA analyses did not substantially alter the main results (Figure S4).

#### 3.3.2 Madeira and the Canary Islands

The RDA of the populations from Madeira and the Canaries showed a structure related to temperature, separating Madeira from the Canary Islands along the first RDA axis, which explained 0.08% of the variation (Figure 3c). This axis was correlated with the annual mean temperature, and individuals from Madeira were negatively correlated with this environmental variable. On the other hand, individuals from La Palma and Gran Canaria showed a separation along the second RDA axis, which was correlated with NDVI and explained 0.03% of the variation (Figure 3c). Individuals from La Palma were positively correlated with NDVI. The pRDA controlling for neutral structure showed that individuals from Madeira did not exhibit a structure related to environmental variables (Figure 3d). However, individuals from La Palma and Gran Canaria showed a clear structure along the first pRDA axis, which was correlated with NDVI and explained 0.03% of the variation (Figure 3d). Individuals from La Palma showed a positive correlation with NDVI, while individuals from Gran Canaria exhibited a negative correlation. Both RDA and pRDA showed statistical significance (*p* < 0.01) in the complete model and in the first two axes.

#### 3.3.3 The Canary Islands

The RDA of the Canarian populations showed a structure separating the western islands (El Hierro and La Palma) from the central islands (La Gomera and Tenerife) along the first RDA axis (Figure 3e). This first axis was correlated with the precipitation of the warmest quarter (PWQ) and explained 0.06% of the variation. Individuals from the western islands showed a positive correlation with precipitation. In addition, individuals from La Palma and Gran Canaria showed a structure along the second RDA axis, which was correlated with NDVI and explained 0.05% of the variation (Figure 3e). Individuals from La Palma were positively correlated with NDVI. On the other hand, the pRDA showed that individuals from Gran Canaria moved to the origin and showed only a relatively minor correlation with PWQ (Figure 3f). Furthermore, individuals from the western islands were separated from individuals from the central islands along the first pRDA axis, which was correlated with PWQ (Figure 3f). This axis explained 0.06% of the variation, and individuals from the western islands showed a positive correlation with precipitation. In addition, this analysis also revealed a structure of individuals from La Palma along the second axis, which was positively correlated with NDVI and explained 0.03% of the variance (Figure 3f). Both RDA and pRDA showed statistical significance (*p* < 0.01) in the complete model in the first two axes.

### 3.4 Identification of Candidate Genes Under Selection

We found that 16 candidate genes were shared between the loci under selection identified by BayeScan and those detected in the RDA analyses, all of which were under purifying selection. Among these, seven were identified in the RDA including all populations, two in the RDA using only the Canary Islands populations, and four in the pRDA of the Canary Islands populations (Table S2).

#### 3.4.1 Candidate Genes in Macaronesia and the Mainland

**FIGURE 4:**
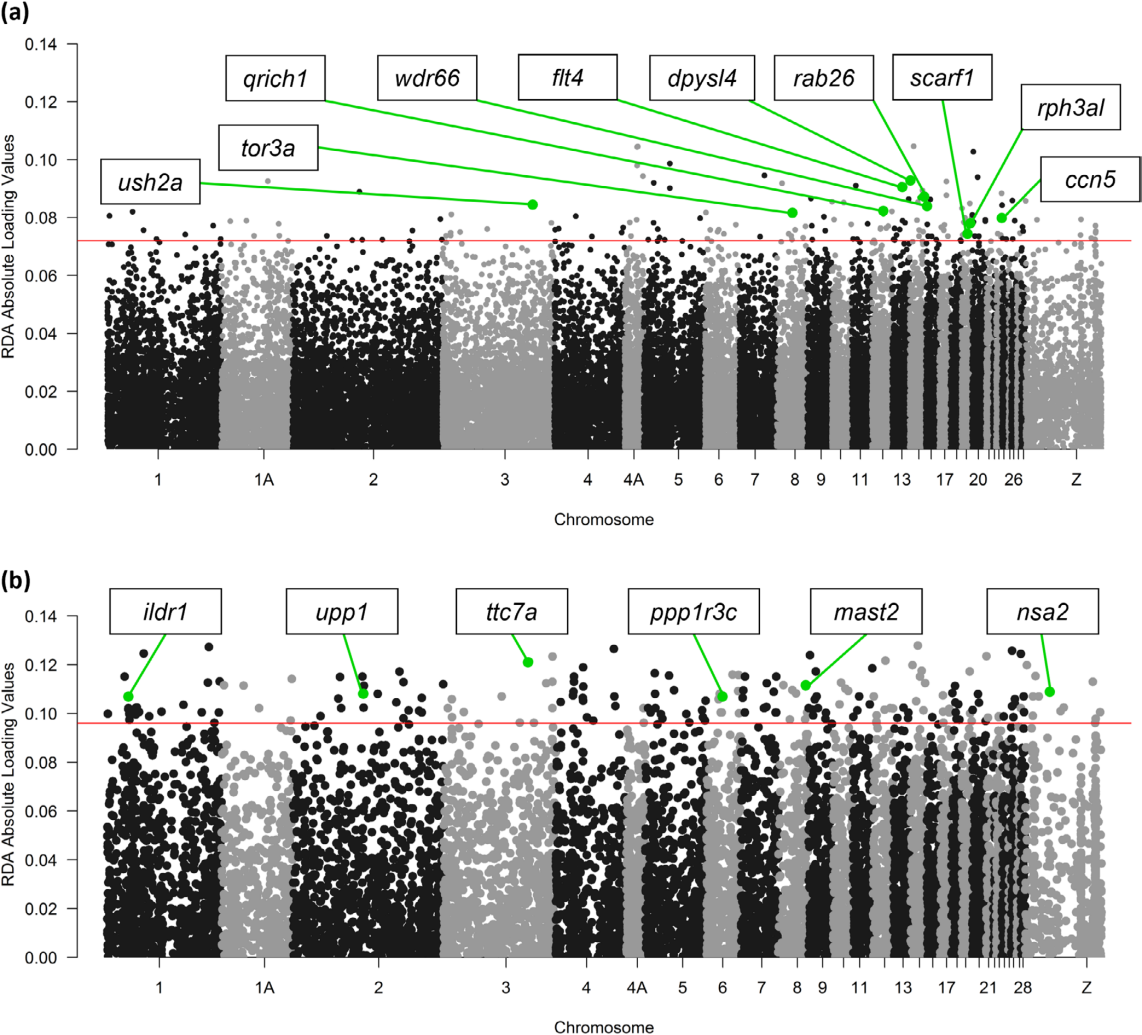
Results of genotype–environment association analyses (GEA) to identify candidate SNPs associated with environmental variables with some of the mapped genes highlighted. Figures represent Manhattan plots conducted with: (a) Absolute values of SNP loadings on partial RDA axis 2 performed using the dataset of all populations, and (b) absolute values of SNP loadings on partial RDA axis 2 conducted using the dataset of Canary Islands populations. The red horizontal lines in (a) represent three standard deviations (SD) from the mean absolute loading value for the analysis, and in (b) they represent 2.5 SD from the mean absolute loading value for the analysis. Green highlighted points represent outlier SNPs associated with candidate genes (labels) related to environmental variables. Black and gray colors distinguish different chromosomes numbered according to the zebra finch reference genome.

The pRDA with the dataset containing all populations from Macaronesia and the mainland revealed 281 outlier SNPs, of which 141 were correlated with NDVI and 140 with precipitation of the driest month (PDM), and mapped onto 50 and 21 genes, respectively (Table S2; Figure 4a). The pRDA found two shared genes between NDVI and PDM (Table S2). We identified 22 (44%) candidate genes associated with phenotypic differences that correlated with NDVI as they are related to known functions such as collagen assembly (*hsd17b12*), development of the skeletal system (*qrich1*, *ccn5*), growth (*brat1*), metabolic pathways (*osbpl5*, *tor3a*), developmental regulation (*tspan6*), inner ear and retinal development (*ush2a*, *cnnm4*), diet and digestive functions (*rab26*), development of the lymphatic and cardiovascular system (*flt4*), and melanocyte differentiation (*onecut2*). In addition, other candidate genes correlated with NDVI were related to nervous system development (*nrp2*, *scarf1*, *dpysl4*, *onecut1*), genetic structure (*usp36*), sperm motility (*wdr66*), and signal transduction (*rph3al*). On the other hand, candidate genes related to PDM are also related to phenotypic differences by being involved in skeletal system development (*pdzrn3*), skeletal muscle system development (*pomgnt1*), keratinocyte differentiation (*prkch*), and metabolic pathways (*dgke*, *rab40c*). Moreover, we identified genes related to telomere elongation (*hmbox1*), microtubule regulation (*mtus2*), nervous system development (*znrf1*), inner ear development (*loxhd1*), and sensory perception (*lrig1*), (Table S2).

#### 3.4.2 Candidate Genes in the Canary Islands

The pRDA performed with the dataset of the Canarian populations detected 184 outlier SNP loci, of which 100 were correlated with NDVI and 84 with the precipitation of the warmest quarter (PWQ), and mapped onto 30 and 24 genes, respectively (Table S2; Figure 4b). The pRDA found two genes shared between NDVI and PWQ; one was the gene *ccser1*, which is related to cellular processes and beak size in other avian species, and the other was the gene *rps3a*, which is related to erythropoiesis (Table S2). The candidate genes associated with NDVI are related to phenotypic traits through known functions such as growth and development of the skeletal system (*qrich1*), striated muscle tissue development (*mrtfb*), development regulation (*wnt7a*), metabolic pathways (*gtpbp2*, *ppp1r3c*, *upp1*), audition (*ildr1*), vision (*gabrr1*), and digestive system development (*ttc7a*). In addition, candidate genes are related to neuronal and nervous system development (*minar1*, *skor2*), genetic and ribosomal structure (*riok1*, *nsa2*), protein structure (*clul1*), transcriptional activity (*ccdc171*), energy homeostasis (*pnpla2*), spermatogenesis (*mast2*), and innate immune response (*nlrp1*, *trim7*). On the other hand, candidate genes related to PWQ are also associated with phenotypic traits by being related to bone system development (*sox5*), cellular structure (*nphp4*), intracellular pH regulation (*slc4a10*), and metabolic pathways (*fah*, *mtmr10*, *fam13a*). Moreover, candidate genes are related to brain and nervous system development (*reln*, *pak3*, *il1rap*) and epigenetic genetic regulation (*dot1l*) (Table S2). Three candidate genes (*nlrp1*, *qrich1*, and *mocs3*) were detected in both the pRDA including all populations and the pRDA with only the Canary Islands. Other candidate genes were unique to each dataset (Table S2).

## 4 Discussion

Our results indicate that Macaronesian chaffinches have diverged from mainland populations and among archipelagos through a combination of adaptive and neutral forces. The SNPs found to be putatively under selection suggest that directional, disruptive selection, and purifying selection have played a role in the Macaronesian chaffinch radiation, with the majority of loci being under purifying selection. Directional/disruptive selection related to resource exploitation within populations is considered a primary factor in adaptive radiations and evolutionary lineage differentiation, as it enables adaptation to local resources (Beausoleil et al. 2019; Gupta and Vadde 2019). Upon colonization of new environments, the exposure of populations to novel selective pressures may result in rapid ecological and phenotypic divergence between populations (Lamichhaney et al. 2015; Martin et al. 2024). Selection may then act on standing genetic variation or de‐novo mutations, which are expected to be localized with peaks of divergence around selected loci, often referred to as “genomic islands of divergence” (Nosil et al. 2009; Martin et al. 2024). However, in small and isolated populations, such as those found on islands, highly divergent genomic regions can also arise, for example, from founder effects (Sendell‐Price et al. 2021) and asymmetric introgression (Bay and Ruegg 2017), and their location can be idiosyncratic, making it essential to interpret genomic divergence cautiously. These highly divergent regions can be responsible for the accumulation of genetic and phenotypic differences between populations, which may play a fundamental role in speciation (Via and West 2008; Martin et al. 2024). The presence of disruptive selection is consistent with the evolutionary history of this species, as the initial phase of Macaronesian colonization involved the colonization of habitats with different selective pressures compared to the continent. However, most of the genes under selection showed low values of *F*_ST_, which indicates the importance of purifying selection in this radiation. Purifying selection is the form of natural selection that acts to eliminate selectively deleterious mutations, and candidate SNPs experiencing stronger negative (purifying) selection exhibit less genetic differentiation between populations, indicating that these loci are experiencing lower evolutionary rates (Hughes et al. 2003; Maruki et al. 2012). In the analysis to identify SNPs putatively under selection using populations from Madeira and Canary Islands, we identified only SNPs under purifying selection, and in the specific analysis of the Canarian populations we did not identify any SNP putatively under selection. These results are consistent with a limited role of positive selection in the differentiation process between islands when habitat differences are relatively minor. However, it is possible that using denser genomic data from whole genomes could reveal regions under selection that were missed with our reduced‐representation method.

The genomic structure revealed by the PCA performed with neutral unlinked SNPs supports the previously established taxonomic differentiation across the study regions (Figure 2a), reflecting the geographical distribution of populations recently reclassified as different species (Recuerda et al. 2021a). The clear structure observed among the five geographic groups highlights the role of historical and geographical factors in shaping genetic divergence of these species. The structure of the Azores chaffinch is consistent with its high genetic diversity values, and with the lowest genetic differentiation from mainland populations in relation to the Macaronesian archipelagos (Recuerda et al. 2021a). The PCA performed with the Canary Islands showed a genomic structure consistent with the populations of the five islands inhabited by the chaffinches (Figure 2b). The populations from island pairs Tenerife and La Gomera, and El Hierro and La Palma, clustered closely, respectively, which is consistent with a relatively recent history of divergence due to geographic proximity (Recuerda et al. 2021a). Finally, the population of Gran Canaria exhibited the greatest genome‐wide differentiation from the other islands, which is also concordant with the longest period of isolation experienced by this population (Suárez et al. 2009; Illera et al. 2018; Recuerda et al. 2021a).

The results obtained in the GEA analyses revealed the relative roles of selection and genetic drift shaping genetic and phenotypic variation between the mainland and Macaronesia, and across the Macaronesian archipelagos. The differentiation between mainland and Macaronesian chaffinches was mainly influenced by local adaptation related to an increase in NDVI, with the exception of the North African species, which primarily differs from the rest due to genetic drift. This is consistent with the vegetation found in the humid laurel forests inhabited by Macaronesian chaffinches, as this habitat exhibits the highest NDVI values, a measure of greenness and vegetation density that effectively distinguishes among different types of vegetation (Pelkey et al. 2003). Habitat type can imply completely different resource availability and be an important factor in local adaptation and possible ecological speciation (Rundle and Nosil 2005). Some studies have reported that the bills of island birds shift in size and shape, reflecting changes in foraging ecology and evolution toward a generalist niche in species‐poor communities (Wright et al. 2016). Island chaffinches show longer, deeper, and wider beaks compared to their mainland counterparts (Grant 1979; Dennison and Baker 1991). Therefore, the larger bill length of Macaronesian chaffinches may provide a selective advantage in conditions that favor a more generalized diet (Grant 1979) as bill morphology is a key trait in local adaptation and differentiation in other taxa (Bosse et al. 2017; Armstrong et al. 2018). Furthermore, we detected selection on the *ccn5* gene related to NDVI, which encodes a member of the wnt1 inducible signaling pathway protein subfamily that has been associated with significant changes in beak morphology and facial morphogenesis in birds (Brugmann et al. 2010; Ji et al. 2014). Both RDA and pRDA models explained a small fraction of the variance, which is expected given the reduced‐representation approach used in this study, as GBS data only capture a small fraction of the genome. This low proportion of variance explained is also likely due to the fact that relatively few SNPs putatively under selection are related to environmental variables and that most traits are likely polygenic, resulting in weaker and more diffuse signals along the genome (Pritchard and Di Rienzo 2010). Nevertheless, the ability of our GEA analyses to detect signals of polygenic selection, consistent with ecological and evolutionary expectations, highlights the robustness and confidence of our results.

Identifying the genetic basis of complex polygenic traits is a challenge even for model species (Pritchard and Di Rienzo 2010; Rockman 2012). In this study, outlier detection methods revealed multiple candidate SNPs distributed across the genome related to environmental and phenotypic traits, as we expected for polygenic traits involved in local adaptation. However, it is also possible that the dispersed signal reflects limitations in statistical power rather than the true genetic architecture of these traits. The overlapping of 16 candidate genes detected by both BayeScan and GEA analyses reinforces the evidence for selection and the strength of our findings, as independent methods identified the same loci under selection. The greater overlap of candidate genes between BayeScan and the RDA using all populations was expected, as neither method explicitly accounts for neutral population structure. The overlapping of some candidate genes across scales suggests that some adaptive processes may be shared across Macaronesia, while unique candidate genes in each dataset may indicate local adaptation to specific environmental conditions. We detected the *nrp2* gene which is related to vocal learning in birds (Cahill et al. 2021), and could be associated with the difference in vocalizations found between mainland and Macaronesian chaffinches, and song divergence among and within archipelagos (Lynch and Baker 1994; Lachlan et al. 2013). Genes related to sperm motility such as *wdr66* (Auguste et al. 2018) could be involved in the significant sperm divergence that exists between different chaffinch species, subspecies, and populations from mainland and Macaronesia (Stensrud 2012). This sperm divergence could drive reproductive incompatibility in the postcopulatory prezygotic phase between different populations (Lifjeld et al. 2016).

Our findings showed that Azores chaffinches exhibited adaptive divergence driven by climatic characteristics. This is consistent with the highest precipitation values reached in Azores compared to the remaining Macaronesian archipelagos (Cropper and Hanna 2014). Outlier detection methods revealed genes (*pdzrn3*) related to precipitation and involved in phenotypic variables such as bone development in birds (Johnsson et al. 2015). The Azores chaffinches have the longest, deepest, widest, and most variable beaks compared to the common chaffinches from the mainland (Grant 1979; Dennison and Baker 1991). Climate and foraging behavior can influence bill size and shape in birds (Beausoleil et al. 2019; Friedman et al. 2019), although it is also possible that resource exploitation competition could explain beak size and shape in the Azores chaffinches (Rando et al. 2010, 2017). Divergence in the Azores is consistent with major adaptive changes occurring in the initial stage of the colonization of Macaronesia, as the morphological difference among the archipelagos is smaller than that between any of the archipelagos and the mainland (Grant 1979). This initial change is too large and complex to be solely explained by neutral forces, although such evolutionary mechanisms also contributed (Grant 1979; Leroy et al. 2021).

Our results suggest that the divergence between North African populations and the remaining chaffinches, as well as between Madeira and the Canary Islands populations, occurred primarily through genetic drift, as genomic variation in these comparisons does not appear to be explained by environmental variables in the pRDA after controlling for neutral genetic structure. Genetic drift, together with sexual selection, was likely involved in driving the marked differences in plumage coloration observed among Macaronesian and North African chaffinches, an important signaling and communication trait (Hill et al. 2006) which could potentially lead to premating reproductive barriers in these taxa (Recuerda et al. 2023). However, this could also reflect the limitations of the environmental variables chosen for analysis, and further studies exploring other potential selective pressures are now needed.

Finally, the analyses of the Canarian populations revealed genetic divergence driven by both neutral and adaptive processes associated with environmental variables. Together with the observed phenotypic divergence among these islands, this suggests an incipient process of differentiation (Illera et al. 2018). The western islands of El Hierro and La Palma exhibited adaptive divergence from the central islands of La Gomera and Tenerife, as their genomic variation is partially explained by precipitation in the pRDA. This is consistent with the precipitation gradient found among the Canary Islands, with the western being wetter than the central islands (Sánchez‐Benítez et al. 2017). Precipitation determines invertebrate prey availability in the Canary Islands (Carrascal et al. 1992; Illera and Díaz 2006), which are a major food source for the Canarian chaffinches during the breeding season (Carrascal et al. 1992; Martín and Lorenzo 2001). Moreover, our results showed that the chaffinches from the laurel forests of La Palma exhibited adaptive divergence from the remaining Canary Islands, with their genomic variation being partly explained by NDVI in the pRDA. This is consistent with the fact that La Palma has the highest area of laurel forest of the Canary Islands (Fernández‐Palacios et al. 2017), which could result in a greater availability of resources in the laurel forests there. A recent study found support for adaptive divergence related to habitat type between the pine and laurel chaffinch populations of La Palma, showing differences in morphology, plumage coloration, and genomic variation (Recuerda et al. 2023). The pRDA of the Canary Islands chaffinches identified genes associated with precipitation such as *ccser1* (also associated with NDVI) and *sox5*, which are related to changes in beak morphology and size in birds (Chavarria‐Pizarro et al. 2019; Yusuf et al. 2020). Importantly, although GBS has limitations as a method of outlier detection, with potential biased estimates of genetic differentiation (Andrews et al. 2016), we were still able to detect signatures of selection. Finally, we showed that individuals from Gran Canaria exhibited the highest genetic divergence from the remaining Canary Islands due to genetic drift, as their genomic variation was not associated with environmental variables after controlling for neutral genetic structure. However, this could also result from selective pressures not captured by our chosen variables. Genetic drift, together with sexual selection, could be shaping the differences in plumage coloration observed among the different subspecies of the Canarian chaffinches.

The radiation experienced by chaffinches in Macaronesia resulted in a limited number of species, contrasting with the higher number of species arising from the iconic radiations of Darwin's finches in Galápagos and the Honeycreepers in Hawaii. These two radiations are considered remarkable examples of how natural selection drives rapid speciation due to ecological specialization. However, radiations can vary in scale and complexity, leading to fewer species, especially when ecological disparity is limited. In the present study, we have shed light on the mechanisms driving differentiation in an avian radiation characterized by harboring few species and a lack of clear ecological specialization. Our findings show that both adaptive and nonadaptive processes shaped the radiation of the chaffinches in the oceanic archipelagos of Macaronesia, revealing candidate genes related to phenotypic traits and environmental variables. Adaptive divergence related to habitat type explained the genetic divergence between mainland and Macaronesian chaffinches, except for African chaffinches, which differed from the rest mainly due to genetic drift. Climate characteristics appear to have driven the divergence of Azores chaffinches, while genetic drift explains the divergence between Madeira and the Canaries. The genetic and morphological variation observed in the Canarian birds was shaped by both adaptive and nonadaptive mechanisms. Whole‐genome studies, with higher sample sizes per population, along with ecological data, are now needed to fully understand the factors behind the radiation observed in the Macaronesian chaffinches.

## Author Contributions

**Brian Condori:** conceptualization (equal), data curation (equal), formal analysis (lead), investigation (supporting), methodology (lead), visualization (lead), writing – original draft (lead), writing – review and editing (equal). **María Recuerda:** conceptualization (equal), data curation (equal), formal analysis (supporting), investigation (equal), methodology (supporting), supervision (supporting), writing – review and editing (equal). **Juan Carlos Illera:** conceptualization (equal), funding acquisition (equal), investigation (equal), resources (equal), supervision (equal), writing – review and editing (equal). **Borja Milá:** conceptualization (equal), funding acquisition (equal), investigation (equal), resources (equal), supervision (equal), writing – review and editing (equal).

## Conflicts of Interest

The authors declare no conflicts of interest.

## Supporting information


Data S1.

## Data Availability

Raw SNP data are deposited at NCBI under the SRA data project PRJNA902955 with accession numbers SRR22329803‐SRR22330002 (see Table S1 for details), and the code and datasets are deposited in Figshare (https://doi.org/10.6084/m9.figshare.28736591.v2). The *Fringilla coelebs* reference genome is deposited at NCBI (Accession number: JADKPM000000000.1).
