# Supplementary material for: The Relative Roles of Selection and Drift in the Chaffinch Radiation (Aves: Fringilla) Across the Atlantic Archipelagos of Macaronesia
Source: Ecol Evol. 2025 Apr 15;15(4):e71307. doi: 10.1002/ece3.71307 (PMC12000227; doi:10.1002/ece3.71307)
Supplement: Supplementary file 1 — Data S1. [file ECE3-15-e71307-s001.docx]

**SUPPLEMENTARY MATERIALS**

**The Relative Roles of Selection and Drift in the Chaffinch Radiation (Aves: Fringilla) Across the Atlantic Archipelagos of Macaronesia**

Brian Condori, María Recuerda, Juan Carlos Illera and Borja Milá

**Genotyping of genome-wide SNP loci**

High quality genomic DNA was extracted from frozen blood preserved in ethanol using a QIAGEN Blood and Tissue kit (Qiagen, Valencia, CA) following the manufacturer’s protocol. SNP discovery was done using GBS (Elshire et al., 2011) from genomic DNA digested with the enzime PstI and sequencing was performed on an Illumina Hiseq X Ten platform. Forward raw reads were trimmed to remove low quality ends using TrimGalore! V, 0.4.4 (<https://www.bioinformatics.babraham.ac.uk/projects/trim_galore/>). Reads were mapped against the latest high-quality version of the chaffinch reference genome (GCA_015532645.2, Recuerda et al., 2021) using the Burrows-Wheeler Aligner [BWA; Li & Durbin, 2009] with the “-mem” algorithm and default parameters. As mean depth coverage was 26.25 (Fig. S1), variant calling was performed with GATK 3.6 HaplotypeCaller and GenotypeGVCFs tools (McKenna et al., 2010), calling all samples together with a minimum base and mapping quality score of 30. The variant dataset obtained was filtered using VCFtools version 0.1.15 (Danecek et al., 2011) keeping biallelic loci with a coverage depth ranging between 5 and 60, a phred quality score over 30, and a minor allele frequency (MAF) over 0.018. Indels were also removed along with sites with over 75% missing data and showing significant deviation from Hardy-Weinberg equilibrium (p-value < 10-4) resulting in a vcf file with 52,306 SNPs. To explore evolutionary processes at different spatial scales, we divided the dataset into multiple subsets. Using VCFtools we created a dataset with only the individuals from Madeira and the Canary Islands, keeping the same parameters as previously used but with a MAF over 0.029. With the same software, another dataset was also created with only the individuals from the Canary Islands, keeping the same parameters as used before but with a MAF over 0.036.

**Genotype-environment association analysis**

To assess the role of selection shaping genomic divergence across different populations, we performed a redundancy analysis (RDA; Legendre & Legendre, 1998) as our GEA method. RDA is an ordination approach which allows estimating the variance in a response variable (here, genomic variation), that can be explained by a set of explanatory variables (here, environmental variables). As a GEA method, allow the detection of weak signals of polygenic selection across the genome and help to identify loci associated with environmental variation. We conducted this analysis using the complete dataset (including neutral SNPs, SNPs under selection, and SNPs without LD pruning) including all populations, the complete dataset with populations from Madeira and the Canary Islands, and the complete dataset only with populations from the Canary Islands. We performed the variable selection using the 'forward.sel' function from the R package 'adespatial' (Dray et al., 2022), which implements an algorithm to select the variables that best explain the spatial structure of the data. The selected variables aligned with our a priori expectations based on known ecological differences between the mainland and Macaronesia, as well as among Macaronesia. As stated in the introduction, habitat type, precipitation, and temperature are key environmental factors distinguishing these regions, and these variables were also identified through our selection procedure. We evaluated the collinearity of the environmental variables ensuring that the selected variables had the lowest Pearson correlation value (|r| <0.7), as recommended by Dormann et al. (2013). We ensured that the variance inflation factor (VIF) of the selected variables was below 10 and a permutation test was performed on the final RDA, as recommended by Borcard et al. (2018). The selected variables were: NDVI and precipitation of the driest month in the analyses with the dataset containing all populations; NDVI and annual mean temperature in the analyses of populations from Madeira and the Canary Islands; and NDVI and precipitation of the warmest quarter in the analyses of the Canarian dataset.

In addition, we performed a partial RDA (pRDA) which allows for the correction of the effects of a set of covariates. In this case, to control for the effect of genetic drift, the pRDA controlled for neutral population structure by including as a covariate the PC1 values from the genomic PCA performed with the datasets that only included neutral SNPs (Forester et al., 2018). Since these values reflected the pattern of isolation by distance, we aimed to avoid incorrect genotype-environment associations due to shared ancestry or geographic isolation (Cao et al., 2019; Gibson & Moyle, 2020; Chang et al., 2022). As the RDA requires datasets without missing data, we imputed missing genotypes (17% in all datasets) using the most common genotype across all individuals in each dataset (Forester et al., 2018). The function used for imputation in R was: “gen.imp <- apply(gen, 2, function(x) replace(x, is.na(x), as.numeric(names(which.max(table(x))))))”. The statistical significance of the complete and per-axis models was tested using ANOVA-like permutation tests setting α=0.01 and using 1,000 permutations. The analyses were conducted using the R package 'vegan' (Oksanen et al., 2022). To evaluate whether incorporating additional PCs would improve control for neutral population structure, we repeated the pRDA analyses including PC1 through PC10 as covariates.

**Tables**

**Table S1.** List of the samples of the different populations and species of the genus *Fringilla* used in the study, including the population to which each sample belongs (POP), NCBI Accession, specimen ID, latitude and longitude.

| **Population** | **Species** | **NCBI Accesion** | **Specimen ID** | **Latitude** | **Longitude** |
| --- | --- | --- | --- | --- | --- |
| Azores | *Fringilla moreletti* | SAMN17349039 | AZ1 | 38.757372 | -27.24940507 |
| Azores | *Fringilla moreletti* | SAMN17349048 | AZ10 | 38.720794 | -27.30933683 |
| Azores | *Fringilla moreletti* | SAMN17349040 | AZ2 | 38.757372 | -27.24940507 |
| Azores | *Fringilla moreletti* | SAMN17349041 | AZ3 | 38.757372 | -27.24940507 |
| Azores | *Fringilla moreletti* | SAMN17349042 | AZ4 | 38.757372 | -27.24940507 |
| Azores | *Fringilla moreletti* | SAMN17349043 | AZ5 | 38.757372 | -27.24940507 |
| Azores | *Fringilla moreletti* | SAMN17349045 | AZ7 | 38.756514 | -27.2458688 |
| Azores | *Fringilla moreletti* | SAMN17349046 | AZ8 | 38.758829 | -27.2506992 |
| Azores | *Fringilla moreletti* | SAMN17349047 | AZ9 | 38.720744 | -27.31439808 |
| Ceuta | *Fringilla spodiogenys* | SAMN17349018 | CEU1 | 35.913281 | -5.362409 |
| Ceuta | *Fringilla spodiogenys* | SAMN17349020 | CEU3 | 35.913281 | -5.362409 |
| Ceuta | *Fringilla spodiogenys* | SAMN17349021 | CEU4 | 35.913281 | -5.362409 |
| Ceuta | *Fringilla spodiogenys* | SAMN17349022 | CEU5 | 35.913281 | -5.362409 |
| Ceuta | *Fringilla spodiogenys* | SAMN17349023 | CEU6 | 35.913281 | -5.362409 |
| Ceuta | *Fringilla spodiogenys* | SAMN17349024 | CEU7 | 35.913281 | -5.362409 |
| Ceuta | *Fringilla spodiogenys* | SAMN17349025 | CEU8 | 35.913281 | -5.362409 |
| Gran Canaria | *Fringilla canariensis bakeri* | SAMN17349058 | GC1 | 28.092961 | -15.59461108 |
| Gran Canaria | *Fringilla canariensis bakeri* | SAMN45034580 | GC10 | 28.073464 | -15.54662996 |
| Gran Canaria | *Fringilla canariensis bakeri* | SAMN17349059 | GC2 | 28.092961 | -15.59461108 |
| Gran Canaria | *Fringilla canariensis bakeri* | SAMN17349060 | GC3 | 28.092961 | -15.59461108 |
| Gran Canaria | *Fringilla canariensis bakeri* | SAMN17349061 | GC4 | 28.092961 | -15.59461108 |
| Gran Canaria | *Fringilla canariensis bakeri* | SAMN17349062 | GC5 | 28.092961 | -15.59461108 |
| Gran Canaria | *Fringilla canariensis bakeri* | SAMN17349063 | GC6 | 28.092961 | -15.59461108 |
| Gran Canaria | *Fringilla canariensis bakeri* | SAMN17349064 | GC7 | 28.092961 | -15.59461108 |
| Gran Canaria | *Fringilla canariensis bakeri* | SAMN17349065 | GC8 | 28.073464 | -15.54662996 |
| La Gomera | *Fringilla canariensis canariensis* | SAMN17349076 | GOM1 | 28.195363 | -17.28771297 |
| La Gomera | *Fringilla canariensis canariensis* | SAMN17349077 | GOM2 | 28.195363 | -17.28771297 |
| La Gomera | *Fringilla canariensis canariensis* | SAMN17349078 | GOM3 | 28.138973 | -17.28419247 |
| La Gomera | *Fringilla canariensis canariensis* | SAMN17349079 | GOM4 | 28.138973 | -17.28419247 |
| La Gomera | *Fringilla canariensis canariensis* | SAMN17349081 | GOM6 | 28.138973 | -17.28419247 |
| El Hierro | *Fringilla canariensis ombriosa* | SAMN17349092 | HI1 | 27.73304 | -18.00986 |
| El Hierro | *Fringilla canariensis ombriosa* | SAMN17349101 | HI10 | 27.73321 | -17.99385 |
| El Hierro | *Fringilla canariensis ombriosa* | SAMN17349093 | HI2 | 27.73343 | -18.00952 |
| El Hierro | *Fringilla canariensis ombriosa* | SAMN17349094 | HI3 | 27.73553 | -17.99816 |
| El Hierro | *Fringilla canariensis ombriosa* | SAMN17349095 | HI4 | 27.73538 | -17.99919 |
| El Hierro | *Fringilla canariensis ombriosa* | SAMN17349096 | HI5 | 27.73469 | -17.99907 |
| El Hierro | *Fringilla canariensis ombriosa* | SAMN17349097 | HI6 | 27.73454 | -18.00006 |
| El Hierro | *Fringilla canariensis ombriosa* | SAMN17349098 | HI7 | 27.73523 | -18.04716 |
| El Hierro | *Fringilla canariensis ombriosa* | SAMN17349099 | HI8 | 27.73523 | -18.04716 |
| El Hierro | *Fringilla canariensis ombriosa* | SAMN17349100 | HI9 | 27.73544 | -17.99326 |
| Madeira | *Fringilla maderensis* | SAMN17349049 | MAD1 | 32.712286 | -16.887391 |
| Madeira | *Fringilla maderensis* | SAMN17349050 | MAD2 | 32.712286 | -16.887391 |
| Madeira | *Fringilla maderensis* | SAMN17349051 | MAD3 | 32.712286 | -16.887391 |
| Madeira | *Fringilla maderensis* | SAMN17349052 | MAD4 | 32.712286 | -16.887391 |
| Madeira | *Fringilla maderensis* | SAMN17349053 | MAD5 | 32.712286 | -16.887391 |
| Madeira | *Fringilla maderensis* | SAMN17349054 | MAD6 | 32.712286 | -16.887391 |
| Madeira | *Fringilla maderensis* | SAMN17349055 | MAD7 | 32.712286 | -16.887391 |
| Madeira | *Fringilla maderensis* | SAMN17349056 | MAD8 | 32.712286 | -16.887391 |
| Madeira | *Fringilla maderensis* | SAMN17349057 | MAD9 | 32.712286 | -16.887391 |
| Segovia | *Fringilla coelebs* | SAMN17349035 | SEG10 | 40.81017 | -4.26463 |
| Segovia | *Fringilla coelebs* | SAMN17349036 | SEG11 | 40.81017 | -4.26463 |
| Segovia | *Fringilla coelebs* | SAMN17349037 | SEG12 | 40.81017 | -4.26463 |
| Segovia | *Fringilla coelebs* | SAMN17349038 | SEG13 | 40.81017 | -4.26463 |
| Segovia | *Fringilla coelebs* | SAMN17349027 | SEG2 | 40.81017 | -4.26463 |
| Segovia | *Fringilla coelebs* | SAMN17349028 | SEG3 | 40.81017 | -4.26463 |
| Segovia | *Fringilla coelebs* | SAMN17349029 | SEG4 | 40.81017 | -4.26463 |
| Segovia | *Fringilla coelebs* | SAMN17349030 | SEG5 | 40.81017 | -4.26463 |
| Segovia | *Fringilla coelebs* | SAMN17349031 | SEG6 | 40.81017 | -4.26463 |
| Segovia | *Fringilla coelebs* | SAMN17349032 | SEG7 | 40.81017 | -4.26463 |
| Segovia | *Fringilla coelebs* | SAMN17349033 | SEG8 | 40.81017 | -4.26463 |
| Segovia | *Fringilla coelebs* | SAMN17349034 | SEG9 | 40.81017 | -4.26463 |
| Tenerife | *Fringilla canariensis canariensis* | SAMN17349067 | TEN1 | 28.321317 | -16.84224217 |
| Tenerife | *Fringilla canariensis canariensis* | SAMN17349068 | TEN2 | 28.321317 | -16.84224217 |
| Tenerife | *Fringilla canariensis canariensis* | SAMN17349069 | TEN3 | 28.321317 | -16.84224217 |
| Tenerife | *Fringilla canariensis canariensis* | SAMN17349071 | TEN5 | 28.52749 | -16.28334 |
| Tenerife | *Fringilla canariensis canariensis* | SAMN17349072 | TEN6 | 28.530718 | -16.27968779 |
| Tenerife | *Fringilla canariensis canariensis* | SAMN17349073 | TEN7 | 28.321317 | -16.84224217 |
| Tenerife | *Fringilla canariensis canariensis* | SAMN17349074 | TEN8 | 28.52749 | -16.28334 |
| Tenerife | *Fringilla canariensis canariensis* | SAMN17349075 | TEN9 | 28.321317 | -16.84224217 |
| La Palma | *Fringilla canariensis palmae* | SRR22329862 | TIL1 | 28.79036 | -17.80168 |
| La Palma | *Fringilla canariensis palmae* | SRR22329852 | TIL2 | 28.79036 | -17.80168 |
| La Palma | *Fringilla canariensis palmae* | SRR22329842 | TIL3 | 28.79036 | -17.80168 |
| La Palma | *Fringilla canariensis palmae* | SRR22329830 | TIL4 | 28.79036 | -17.80168 |
| La Palma | *Fringilla canariensis palmae* | SRR22329828 | TIL5 | 28.79036 | -17.80168 |
| La Palma | *Fringilla canariensis palmae* | SAMN45034581 | TIL6 | 28.79036 | -17.80168 |
| La Palma | *Fringilla canariensis palmae* | SRR22329827 | TIL7 | 28.79036 | -17.80168 |
| La Palma | *Fringilla canariensis palmae* | SRR22329826 | TIL8 | 28.79036 | -17.80168 |
| La Palma | *Fringilla canariensis palmae* | SRR22329825 | TIL81 | 28.79036 | -17.80168 |
| La Palma | *Fringilla canariensis palmae* | SRR22329824 | TIL82 | 28.79036 | -17.80168 |

**Table S2.** Candidate genes identified in the GBS dataset through different analyses: (a) RDA performed using the dataset with all populations, (b) Partial RDA performed using the dataset with all populations, (c) RDA performed using the dataset with populations from Madeira and the Canary Islands, (d) Partial RDA performed using the dataset with populations from Madeira and the Canary Islands, (e) RDA performed using the dataset with the Canary Islands, (f) Partial RDA performed using the dataset with the Canary Islands, and (g) BayeScan using all populations.

| **Gene** | **Environmental variable and analysis** | **Name** | **Pathways** | **Gene ontology** |
| --- | --- | --- | --- | --- |
| PDP2 | NDVI  (a,b,c) | Pyruvate Dehydrogenase Phosphatase Catalytic Subunit 2 | Pyruvate metabolism and respiratory electron transport | Serine/Threonine Protein Phosphatase Activity |
| PDP1 | NDVI  (a,b,c) | Pyruvate Dehydrogenase Phosphatase Catalytic Subunit 1 | Pyruvate metabolism and respiratory electron transport | Serine/Threonine Protein Phosphatase Activity |
| RHCG | NDVI (a) | Rhesus Blood Group Family Type C Glycoprotein | Transport of inorganic cations/anions and amino acids/oligopeptides | Transmembrane Ammonium Transporter Activity and Ankyrin Binding |
| CCN5 (CTGF-L) | NDVI (a,b,d) | Cellular Communication Network Factor 5 | WNT1-Inducible Signaling Pathway (WISP) | Plays an important role in bone turnover modulation. It promotes osteoblast cell adhesion and inhibits fibrinogen binding to integrin receptors |
| TSPAN6 | NDVI (a,b,c,d)  PWQ (e) | Tetraspanin 6 | Negative regulator of retinoic acid-inducible gene I-like receptor-mediated immune signaling | It is involved in the regulation of cell development, activation, growth, and motility |
| TSPAN7 | NDVI (a,b,c,d)  PWQ (e) | Tetraspanin 7 | Transmission across chemical synapses and trafficking of AMPA receptors | Regulation of cellular development, activation, growth, and motility |
| COL26A1 | NDVI (a) (g) | Collagen Type XXVI Alpha 1 Chain | Trimerization of the collagen chain and organization of the extracellular matrix | Collagen development and extracellular matrix organization |
| SH2D3C | NDVI (a,b) | SH2 Domain Containing 3C | BCAR1-CRK-RAPGEF1 signaling and activation of the small GTPase RAP1 | Cell adhesion and migration, tissue organization, and regulation of immune response |
| GPSM1 | NDVI (a) | G Protein Signaling Modulator 1 | Downstream signaling of GPCR and signal transduction | Development of the nervous system |
| QRICH1 | NDVI (a,b,c,d,e,f) | Glutamine Rich 1 | CASP3/caspase-3 activity in epithelial cells under stress | It is involved in chondrocyte hypertrophy, a necessary process for normal longitudinal bone growth |
| MOCS3 | NDVI (a,b,c,d,f) | Molybdenum Cofactor Synthesis 3 | Metabolism of water-soluble vitamins and cofactors and glycolysis | Nucleotidyltransferase activity and sulfurtransferase activity |
| PLB1 | NDVI (a) | Phospholipase B1 | Visual phototransduction and glycerophospholipid biosynthesis | Digestion of phospholipids, glycerolipids, and dietary retinoids, and facilitation of lipid absorption. |
| SUFU | NDVI (a) (g) | SUFU Negative Regulator Of Hedgehog Signaling | Negative regulation of the Hedgehog signaling pathway | Plays an important role in early human development |
| BRAT1 | NDVI (a,b,c,e) | BRCA1 Associated ATM Activator 1 | Cell cycle checkpoint signalling pathways | Required for cell cycle progress by growth factors |
| HSD17B12 | NDVI (a,b)  PWQ (e) | Hydroxysteroid 17-Beta Dehydrogenase 12 | Fatty acyl-CoA biosynthesis and Metabolism | Oxidoreductase activity and collagen binding |
| CDKL1 | NDVI (a) | Cyclin Dependent Kinase Like 1 | Serine/threonine protein kinases | Transferase activity, transfer of phosphorus-containing groups, and protein tyrosine kinase activity |
| PAX4 | NDVI (a,d)  PDM (a) (g) | Paired Box 4 | Embryonic and Induced Pluripotent Stem Cells and Lineage-specific Markers | Fetal development, development of the endocrine system (insulin-secreting beta cells) |
| PAK3 | NDVI (a)  PWQ (e,f) (g) | P21 (RAC1) Activated Kinase 3 | Signaling by Rho GTPases and the integrin pathway | Development of the nervous system, dendrite morphogenesis, as well as in synapse formation and plasticity |
| RNF145 | PDM (a) (g) | Ring Finger Protein 145 | Promotes the monoubiquitination of CYBA | It is involved in maintaining cholesterol homeostasis |
| ZMYND12 | PDM (a) | Zinc Finger MYND-Type Containing 12 |  | It is predicted to enable metal ion binding activity |
| SRGAP2 | PDM (a) (g) | SLIT-ROBO Rho GTPase Activating Protein 2 | Signaling by Rho GTPases | Development of the nervous system |
| PPP1R12B | PDM (a) (g) | Protein Phosphatase 1 Regulatory Subunit 12B | Beta-Adrenergic Signaling and Activation of cAMP-Dependent PKA | It regulates the activity of myosin phosphatase and increases the sensitivity to Ca(2+) of the contractile apparatus |
| COL27A1 | PDM (a) | Collagen Type XXVII Alpha 1 Chain | Collagen chain trimerization and Integrin Pathway | It is involved in cartilage calcification and in the transition from cartilage to bone |
| DLGAP2 | PDM (a) (g) | DLG Associated Protein 2 | Adapter protein linking ion channel to the subsynaptic cytoskeleton | Synapse organization and signalling in neuronal cells |
| FLT4 | NDVI (b) | Fms Related Receptor Tyrosine Kinase 4 | Apoptotic Pathways in Synovial Fibroblasts and GPCR Pathway | Development of the lymphatic system and development of the cardiovascular system in embryonic development |
| SGCZ | NDVI (b) | Sarcoglycan Zeta | Dystrophin-associated glycoprotein complex (DGC) | Maintenance of striated muscle membrane stability |
| NDFIP1 | NDVI (b) | Nedd4 Family Interacting Protein 1 | AKT1-to-MAPK8 signaling in response to EGF | Obsolete signaling transduction activity and binding to WW domains |
| DPYSL4 | NDVI (b) | Dihydropyrimidinase Like 4 | Nervous system development and Semaphorin interactions | Development of the nervous system |
| DPYSL3 | NDVI (b) | Dihydropyrimidinase Like 3 | Nervous system development and Semaphorin interactions | Development of the nervous system |
| FER | NDVI (b)  PDM (b) | FER Tyrosine Kinase | Immune response Fc epsilon RI pathway and Signaling by SCF-KIT | Transferase activity, transfer of phosphorus-containing groups, and protein tyrosine kinase activity |
| FES | NDVI (b)  PDM (b) | FES Proto-Oncogene, Tyrosine Kinase | Semaphorin interactions and Nervous system development | Regulation of cell differentiation and promotion of neurite growth |
| INTS14 | NDVI (b) | Integrator Complex Subunit 14 | Gene expression (Transcription) and Formation of HIV complex | Transcription of small nuclear RNAs |
| GNB5 | NDVI (b) | G Protein Subunit Beta 5 | ADORA2B anti-inflammatory cytokines production | Neuronal signaling |
| NRP2 | NDVI (b) | Neuropilin 2 | Signaling receptor activity and growth factor binding | Development of the nervous system |
| USP36 | NDVI (b) | Ubiquitin Specific Peptidase 36 | Ubiquitin-Proteasome Dependent Proteolysis | Regulation of nucleolar structure and function |
| LGR6 | NDVI (b) | Leucine Rich Repeat Containing G Protein-Coupled Receptor 6 | WNT signaling and ncRNAs involved in Wnt signaling in hepatocellular carcinoma | It can act as a tumor suppressor |
| LMNA | NDVI (b,d) | Lamin A/C |  | Nuclear stability, chromatin structure, and gene expression |
| LMNB1 | NDVI (b,d) | Lamin B1 |  | Nuclear stability, chromatin structure, and gene expression |
| LMNB2 | NDVI (b,d) | Lamin B2 |  | Nuclear stability, chromatin structure, and gene expression |
| FRAS1 | NDVI (b) | Fraser Extracellular Matrix Complex Subunit 1 | Integrin Pathway and ERK Signaling | Brain organization and function |
| WDR66 | NDVI (b) | Cilia And Flagella Associated Protein 251 (CFAP251) |  | Sperm motility |
| RNF185 | NDVI (b,c,d) | Ring Finger Protein 185 | Calnexin/calreticulin cycle | Ligase activity |
| FAM122A | NDVI (b) | PP2A Aalpha (PPP2R1A) And B55A (PPP2R2A) Interacting Phosphatase Regulator 1 | Serine/threonine-protein phosphatase 2A (PP2A) activity | Positive regulation of cell growth |
| FAM122B | NDVI (b) | PABIR Family Member 2 | Inhibitory activity of serine/threonine protein phosphatase | Negative regulation of catalytic activity and part of the collagen trimer |
| NLRP1 | NDVI (b,c,d,e,f) | NLR Family Pyrin Domain Containing 1 | Innate Immune System and Inflammasomes | Critical functions in innate immunity and inflammation |
| EIF2A | NDVI (b) | Eukaryotic Translation Initiation Factor 2A | Translation initiation factor activity | Ribosome binding |
| SCARF1 | NDVI (b) | Scavenger Receptor Class F Member 1 | Binding and Uptake of Ligands by Scavenger Receptors and Vesicle-mediated transport | Regulation of neurite growth, nervous system |
| RPH3AL | NDVI (b) | Rabphilin 3A Like (Without C2 Domains) | Dysregulation of Rab genes and Rab effectors in bladder cancer | Insulin secretion by pancreatic cells, regulator of exocytosis |
| CEP250 | NDVI (b) | Centrosomal Protein 250 | Loss of Nlp from mitotic centrosomes and cell cycle | Centrosome cohesion during interphase |
| USH2A | NDVI (b) | Usherin | Collagen binding and myosin binding | Development and homeostasis of the inner ear and retina |
| PRDX3 | NDVI (b) | Peroxiredoxin 3 | Cellular responses to stimuli and nuclear events mediated by NFE2L2 | Cellular protection against oxidative stress through peroxide detoxification |
| CNNM2 | NDVI (b) | Cyclin and CBS Domain Divalent Metal Cation Transport Mediator 2 | Adenyl nucleotide binding | Magnesium homeostasis by mediating epithelial transport and renal reabsorption of Mg2+ |
| CNNM4 | NDVI (b) | Cyclin and CBS Domain Divalent Metal Cation Transport Mediator 4 | Adenyl nucleotide binding | It may play a role in biomineralization and retinal function |
| RAB26 | NDVI (b) | RAB26, Member RAS Oncogene Family | Protein metabolism and dynamics of Sertoli-Sertoli cell junctions | Secretion of pepsinogen and amylase in digestion, digestive function |
| ANKS3 | NDVI (b) | Ankyrin Repeat And Sterile Alpha Motif Domain Containing 3 | Ciliary landscape and Ciliopathies | May be involved in vasopressin signaling in the kidney |
| ATF7IP | NDVI (b) | Activating Transcription Factor 7 Interacting Protein | Chromatin organization and PKMTs methylate histone lysines | Regulation of chromatin formation |
| SBF2 | NDVI (b) | SET Binding Factor 2 | PI Metabolism and Vesicle-mediated transport | Homodimerization activity and binding to phosphatidylinositol |
| OSBPL5 | NDVI (b) | Oxysterol Binding Protein Like 5 | Biosynthesis and metabolism of glycerophospholipids | Regulation of cholesterol |
| CD80 | NDVI (b) | CD80 Molecule | MIF Mediated Glucocorticoid Regulation and TGF-Beta Pathway | Coreceptor activity |
| TOR3A | NDVI (b) | Torsin Family 3 Member A | Metabolic pathways | ATP hydrolysis activity |
| SLC6A9 | NDVI (b) | Solute Carrier Family 6 Member 9 | Transport of inorganic cations/anions and amino acids/oligopeptides | Modulation of the nervous system |
| ONECUT1 | NDVI (b) | One Cut Homeobox 1 | Regulation of transcription by RNA polymerase II | Regulation of beta-cell development and nervous system development |
| ONECUT2 | NDVI (b) | One Cut Homeobox 2 | Embryonic and Induced Pluripotent Stem Cells and Lineage-specific Markers | The protein binds to specific DNA sequences and stimulates the expression of target genes, including genes involved in melanocyte and hepatocyte differentiation |
| ONECUT3 | NDVI (b) | One Cut Homeobox 3 | Regulation of transcription by RNA polymerase II | Regulation of beta-cell development and nervous system development |
| HBEGF | PDM (b) | Heparin Binding EGF Like Growth Factor | Apoptotic Pathways in Synovial Fibroblasts and Signaling by ERBB2 KD Mutants | Enables growth factor activity and heparin-binding activity |
| ZNRF1 | PDM (b) | Zinc And Ring Finger 1 | Class I MHC mediated antigen processing and presentation | Development of the nervous system, neuron differentiation |
| DGKE | PDM (b) | Diacylglycerol Kinase Epsilon | GPCR downstream signalling and Response to elevated platelet cytosolic Ca2+ | Regulates the respective levels of bioactive lipids |
| BPTF | PDM (b) | Bromodomain PHD Finger Transcription Factor | 16p11.2 proximal deletion syndrome and Endoderm differentiation | Regulation of transcription |
| SUCLG1 | PDM (b) | Succinate-CoA Ligase GDP/ADP-Forming Subunit Alpha | TCA cycle III (animals) and respiratory electron transport in ATP synthesis | RNA binding and GTP binding |
| RNF34 | PDM (b) | Ring Finger Protein 34 | Gene expression (Transcription) and Class I MHC mediated antigen processing and presentation | Ligase activity and ubiquitin ligase protein binding |
| LRIG1 | PDM (b) | Leucine Rich Repeats And Immunoglobulin Like Domains 1 | Signaling by EGFR and MET promotes cell motility. | Innervation, otolith morphogenesis, and sensory and sound perception. Feather development in avian studies |
| PDZRN3 | PDM (b) | PDZ Domain Containing Ring Finger 3 | Regulation of surface level of MUSK in myotubes | Vascular development and differentiation of adipocytes, osteoblasts, and myoblasts |
| PDZRN4 | PDM (b) | PDZ Domain Containing Ring Finger 4 | Ubiquitin-protein transferase activity and ubiquitin-protein ligase activity | Enables metal ion binding activity |
| HMBOX1 | PDM (b) | Homeobox Containing 1 | miR-222 in exercise-induced cardiac growth | Exercise-induced cardiac growth and alternative lengthening of telomeres (ALT) in telomerase-negative cells |
| RAB40C | PDM (b) | RAB40C, Member RAS Oncogene Family | Metabolism of proteins and Autophagy pathway | GTP binding and GDP binding |
| ARFGAP2 | PDM (b) | ADP Ribosylation Factor GTPase Activating Protein 2 | Transport to the Golgi and subsequent modification and Golgi-to-ER retrograde transport | Enables GTPase activation activity |
| PRKCH | PDM (b) | Protein Kinase C Eta | Prolactin Signaling and ADORA2B mediated anti-inflammatory cytokines production | Regulation of keratinocyte differentiation |
| APBB1IP | PDM (b) | Amyloid Beta Precursor Protein Binding Family B Member 1 Interacting Protein | Signaling downstream of RAS mutants and RAF/MAP kinase cascade | Signal transduction |
| MTUS2 | PDM (b) | Microtubule Associated Scaffold Protein 2 | Together with MAPRE1, it can direct the microtubule depolymerase KIF2C to the positive end of microtubules | Regulation of microtubules at their distal growing end |
| CRIP2 | PDM (b) | Cysteine Rich Protein 2 | VEGFA-VEGFR2 signaling | Differentiation of smooth muscle tissue |
| POMGNT1 | PDM (b) | Protein O-Linked Mannose N-Acetylglucosaminyltransferase 1 (Beta 1,2-) | O-linked glycosylation of mucins and Metabolism of proteins | Regulatory activity and muscle development in birds |
| CTIF | PDM (b) | Cap Binding Complex Dependent Translation Initiation Factor | RNA binding and binding | Plays a central role in mRNA decay |
| LOXHD1 | PDM (b) | Lipoxygenase Homology PLAT Domains 1 | Calcium channel activity | Necessary for the normal function of hair cells in the inner ear |
| CENPT | NDVI (c,d) | Centromere Protein T | EML4 and NUDC in mitotic spindle formation and Separation of Sister Chromatids | Kinetochore assembly, chromosome organization, and normal progression of mitosis |
| NECAB2 | NDVI (c,d) | N-Terminal EF-Hand Calcium Binding Protein 2 | Can modulate ligand-induced internalization of ADORA2A and coupling efficiency of mGluR5/GRM5 | Neural modulation |
| NFKB1 | NDVI (c,d) | Nuclear Factor Kappa B Subunit 1 | MyD88 dependent cascade initiated on endosome and TNFR1 Pathway | Biological processes such as inflammation, immunity, differentiation, cell growth, tumorigenesis, and apoptosis |
| RPS3A | NDVI (c,d,f)  PWQ (f) | Ribosomal Protein S3A | Peptide chain elongation and Activation of the mRNA upon binding of the cap-binding complex and eIFs | Erythropoiesis |
| ZBTB49 | NDVI (c) | Zinc Finger And BTB Domain Containing 49 | Activation of CDKN1A/p21 or RB1 transcription | Binding of nucleic acids and transcription factors |
| MED15 | NDVI (c) | Mediator Complex Subunit 15 | Gene expression (Transcription) and PPARA activates gene expression | Transcription regulation, genetic regulation of cholesterol |
| FLT1 | NDVI (c,d) | Fms Related Receptor Tyrosine Kinase 1 | Apoptotic Pathways in Synovial Fibroblasts and GPCR Pathway | Development of embryonic vasculature, regulation of angiogenesis, cell survival, cell migration |
| RNF13 | NDVI (c,d) | Ring Finger Protein 13 | Protein-protein interactions | Ligase activity and regulation of cell proliferation |
| FLOT2 | NDVI (c) | Flotillin 2 | Signaling by Rho GTPases and RHOC GTPase cycle | Neural signalling |
| CPM | NDVI (c,d) | Carboxypeptidase M | Metabolism of proteins and Post-translational modification: synthesis of GPI-anchored proteins | Control of peptide hormone and growth factor activity on the cell surface |
| FUBP3 | NDVI (c) | Far Upstream Element Binding Protein 3 | Positive regulation of transcription by RNA polymerase II | Nucleic acid and RNA binding |
| PRKCD | NDVI (c) | Protein Kinase C Delta | Prolactin Signaling and Calmodulin induced events | Transfer of phosphorus-containing groups and protein tyrosine kinase activity |
| CHD6 | NDVI (c,d) | Chromodomain Helicase DNA Binding Protein 6 | Transcription of specific genes in response to oxidative stress through interaction with NFE2L2 | Chromatin remodeling |
| CTNNBL1 | NDVI (c,d,e,f) | Catenin Beta Like 1 | Processing of Capped Intron-Containing Pre-mRNA | Enzyme binding |
| NOL4 | NDVI (c) | Nucleolar Protein 4 |  | RNA binding |
| ZNF511 | NDVI (c,e,f) | Zinc Finger Protein 511 | RNA polymerase II activity | Regulation of transcription |
| ADAP1 | NDVI (c) (g) | ArfGAP With Dual PH Domains 1 | Signaling by ERBB4 and B Cell Receptor Signaling Pathway | Metabolic pathways |
| PNPLA2 | NDVI (c,d) | Patatin Like Phospholipase Domain Containing 2 | Glycerophospholipid biosynthesis and Regulation of Insulin-like Growth Factor (IGF) transport and uptake by Insulin-like Growth Factor Binding Proteins (IGFBPs) | Energetic homeostasis, enhancing triglyceride hydrolysis and providing fatty acids in response to starvation |
| PSMA3 | NDVI (c,d,e,f) | Proteasome 20S Subunit Alpha 3 | Regulation of activated PAK-2p34 | Maintenance of protein homeostasis |
| SPIRE1 | NDVI (c,d) | Spire Type Actin Nucleation Factor 1 | TGF-Beta Pathway and VEGFA-VEGFR2 signaling | Intracellular transport |
| ILDR1 | NDVI (c,d,f) | Immunoglobulin Like Domain Containing Receptor 1 | Regulates alternative splicing of pre-mRNA by binding to TRA2A, TRA2B, and SRSF1 | Critical for normal hearing by maintaining the structural and functional integrity of tTJs (tight junctions) |
| PLA1A | NDVI (c,d) | Phospholipase A1 Member A | Glycerophospholipid biosynthesis and Metabolism | Hydrolase activity and phospholipase A1 activity |
| TM4SF19 | NDVI (c) | Transmembrane 4 L Six Family Member 19 | 3q29 copy number variation syndrome | Cell proliferation and motility and adhesion via interactions with integrins |
| OFD1 | NDVI (c) | OFD1 Centriole And Centriolar Satellite Protein | Loss of Nlp from mitotic centrosomes and Cell Cycle, Mitotic | Cell development |
| GFI1 | NDVI (c) | Growth Factor Independent 1 Transcriptional Repressor | RNA Polymerase I Promoter Opening and Nervous system development | Cellular development, hematopoiesis, and nervous system development |
| OLFML2B | NDVI (c,d) | Olfactomedin Like 2B | Extracellular matrix binding | Cellular reorganisation |
| MAST2 | NDVI (c,d,e,f) | Microtubule Associated Serine/Threonine Kinase 2 | Une la red distrofina/utrofina con los filamentos de microtúbulos vía sintrofinas | Spermatid maturation, cellular reorganization, and structuring |
| PTPRD | NDVI (c,d) | Protein Tyrosine Phosphatase Receptor Type D | Protein-protein interactions at synapses and Transmission across Chemical Synapses | Cell growth, differentiation, and presynaptic differentiation |
| C6 | NDVI (c,d) | Complement C6 | Complement cascade and Immune response Lectin induced complement pathway | Innate immune response |
| NEFL | NDVI (d) | Neurofilament Light Chain | RAF/MAP kinase cascade and Unblocking of NMDA receptors, glutamate binding and activation | Neural structuring |
| BTBD10 | NDVI (d)  PWQ (f) (g) | BTB Domain Containing 10 | Activation of AKT family members by inhibiting PPP2CA-mediated dephosphorylation | Neuronal maintenance and acceleration of pancreatic beta cell growth |
| KCTD20 | NDVI (d)  PWQ (f) (g) | Potassium Channel Tetramerization Domain Containing 20 |  | Identical protein binding activity, positive regulation of phosphorylation, integral membrane component |
| KDR | NDVI (d) | Kinase Insert Domain Receptor | Apoptotic Pathways in Synovial Fibroblasts and GPCR Pathway | Endothelial cell growth factor |
| MAPKAPK5 | NDVI (d) | MAPK Activated Protein Kinase 5 | Gene expression (Transcription) and Regulation of TP53 Activity. | Cell signalling |
| TPRN | NDVI (d) | Taperin | Sensory processing of sound and Olfactory Signaling Pathway | Sound processing and olfactory pathway signalling |
| NDRG3 | NDVI (d) | NDRG Family Member 3 | PI3K / Akt Signaling | Angiogenesis (CST), apoptosis, and autophagy |
| CCNDBP1 | NDVI (d) | Cyclin D1 Binding Protein 1 | Inhibition of the cyclin-D1/CDK4 complex | Cellular development, signaling in leukocytes |
| CABLES2 | NDVI (d) | Cdk5 And Abl Enzyme Substrate 2 | Response to elevated platelet cytosolic Ca2+ and Golgi-to-ER retrograde transport | Cell division and cell cycle regulation |
| AKT3 | NDVI (d) | AKT Serine/Threonine Kinase 3 | IL-9 Signaling Pathways and Translation Insulin regulation of translation | Metabolism, proliferation, cell survival, growth, and angiogenesis |
| RHBDF1 | NDVI (d)  PWQ (f) | Rhomboid 5 Homolog 1 | Serine-type endopeptidase activity and binding to growth factor | It is involved in sleep, cell survival, proliferation, migration, and inflammation |
| TRIM7 | AMT (d)  NDVI (f) | Tripartite Motif Containing 7 | MEK/ERK pathway and regulation of JUN transactivation | Innate immunity, cell proliferation, and migration |
| POLR1B | NDVI (e) | RNA Polymerase I Subunit B | RNA Polymerase I Promoter Opening and RNA Polymerase I Transcription Termination | Transcription of ribosomal RNA (rRNA) genes and rRNA production |
| FAH | PWQ (e,f) | Fumarylacetoacetate Hydrolase | Tyrosine degradation and Metabolism | Endocrine function |
| SLC4A10 | PWQ (e,f) | Solute Carrier Family 4 Member 10 | Transport of inorganic cations/anions and amino acids/oligopeptides | Intracellular pH regulation |
| DOT1L | PWQ (e,f) | DOT1 Like Histone Lysine Methyltransferase | PKMTs methylate histone lysines and Chromatin organization | Key role in epigenetic genetic regulation |
| E4F1 | PWQ (e,f) | E4F Transcription Factor 1 | p53 pathway | Cell survival and proliferation through cell cycle control |
| RELN | PWQ (e,f) | Reelin | PI3K-Akt signaling pathway | Brain and nervous system development |
| SOX5 | PWQ (e,f) | SRY-Box Transcription Factor 5 | Endochondral ossification with skeletal dysplasias and ERK Signaling | Regulation of cartilage formation during embryonic development |
| VILL | PWQ (e,f) | Villin Like | Actin binding, structural constituent of the cytoskeleton | Cell structuring |
| CCDC171 | PWQ (e,f) (g) | Coiled-Coil Domain Containing 171 |  | DNA-binding transcription factor and signal transduction activity |
| ZNF319 | NDVI (f) | Zinc Finger Protein 319 | Regulation of transcription by RNA polymerase II | Regulation of transcription |
| MINAR1 | NDVI (f) | Membrane Integral NOTCH2 Associated Receptor 1 | Negative regulation of mTOR signaling pathway | Neuronal development and angiogenesis |
| CCSER1 | NDVI (f)  PWQ (f) | Coiled-Coil Serine Rich Protein 1 |  | Cellular processes. Involved in bill size in *Setophaga petechia xanthotera* |
| WNT7A | NDVI (f) | Wnt Family Member 7A | ncRNAs involved in Wnt signaling in hepatocellular carcinoma and the Wnt pathway | Embryonic development, including dorsal versus ventral patterning during limb development, skeleton development, and urogenital tract development |
| WNT7B | NDVI (f) | Wnt Family Member 7B | ncRNAs involved in Wnt signaling in hepatocellular carcinoma and the Wnt pathway | Developmental processes, such as regulation of cell fate and pattern during embryogenesis |
| GABRR1 | NDVI (f) | Gamma-Aminobutyric Acid Type A Receptor Subunit Rho1 | Transmission through chemical synapses and activation of the GABA B receptor | Retinal neurotransmission |
| GTPBP2 | NDVI (f) | GTP Binding Protein 2 | Response to elevated platelet cytosolic Ca2+ and Autophagy | Metabolic pathways |
| TTC7A | NDVI (f) | Tetratricopeptide Repeat Domain 7A | Central role in the binding of PI4KA to EFR3B and HYCC1 | Intestinal development |
| VRK2 | NDVI (f) | VRK Serine/Threonine Kinase 2 | Cell Cycle, Mitotic and Signaling by Rho GTPases | Metabolic pathways |
| PPP1R3C | NDVI (f) | Protein Phosphatase 1 Regulatory Subunit 3C | Beta-Adrenergic Signaling and Activation of cAMP-Dependent PKA | Metabolic pathways |
| PPP1R3E | NDVI (f) | Protein Phosphatase 1 Regulatory Subunit 3E | ER stress and Beta-Adrenergic Signaling | Metabolic pathways |
| OIT3 | NDVI (f) | Oncoprotein Induced Transcript 3 | Binding of calcium ions | Function and hepatocellular development |
| MRTFB | NDVI (f) | Myocardin Related Transcription Factor B | Acts as a transcriptional coactivator of the serum response factor (SRF) | Development of striated muscle tissue |
| SPTB | NDVI (f) | Spectrin Beta, Erythrocytic | Transport to the Golgi and subsequent modification and RAF/MAP kinase cascade | Structure of erythrocytes |
| CLUL1 | NDVI (f) | Clusterin Like 1 |  | Protein structuring |
| UPP1 | NDVI (f) | Uridine Phosphorylase 1 | Nucleotide salvage and Fluoropyrimidine activity | Metabolic pathways |
| RIOK1 | NDVI (f) | RIO Kinase 1 | rRNA processing in the nucleus and cytosol and Processing of Capped Intron-Containing Pre-mRNA | Nucleic acid structuring |
| SKOR2 | NDVI (f) | SKI Family Transcriptional Corepressor 2 | Negative regulation of the transforming growth factor beta receptor signaling pathway | Nervous system development |
| CCDC152 | NDVI (f) | Coiled-Coil Domain Containing 152 |  | Enables protein binding |
| NSA2 | NDVI (f) | NSA2 Ribosome Biogenesis Factor | Biogenesis of the 60S ribosomal subunit | rRNA structuring |
| MTMR10 | PWQ (f) | Myotubularin Related Protein 10 | PI Metabolism and Metabolism | Metabolic pathways |
| CARF | PWQ (f) | Calcium Responsive Transcription Factor | DNA-binding transcription factor activity | Positive regulation of transcription |
| NPHP4 | PWQ (f) | Nephrocystin 4 | Organelle biogenesis and maintenance and Loss of Nlp from mitotic centrosomes | Structural molecule activity. |
| FAM13A | PWQ (f) | Family With Sequence Similarity 13 Member A | Signaling by Rho GTPases and RAC1 GTPase cycle | Metabolic pathways |
| IL1RAP | PWQ (f) | Interleukin 1 Receptor Accessory Protein | IL-1 Family Signaling Pathways and Bacterial infections in CF airways | Development of the nervous system |
| TRIM37 | PWQ (f) | Tripartite Motif Containing 37 | R Class I MHC mediated antigen processing and presentation and Innate Immune System | Transcription regulation, cellular development |
| GID4 | PWQ (f) | GID Complex Subunit 4 Homolog | Smith-Magenis and Potocki-Lupski syndrome copy number variation and Ciliary landscape | Activation of RNA polymerase II transcription |
| NSUN2 | PWQ (f) | NOP2/Sun RNA Methyltransferase 2 | tRNA processing and Processing of Capped Intron-Containing Pre-mRNA | Growth and cell proliferation |
| SLC25A36 | PWQ (f) | Solute Carrier Family 25 Member 36 | Pyrimidine nucleotide transmembrane transporter activity | Mitochondrial genome maintenance, regulation of mitochondrial membrane potential and mitochondrial respiration |
| ADAMTS12 | PWQ (f) | ADAM Metallopeptidase With Thrombospondin Type 1 Motif 12 | O-linked glycosylation of mucins and Metabolism of proteins | Regulation of cell adhesion |

**Figures**


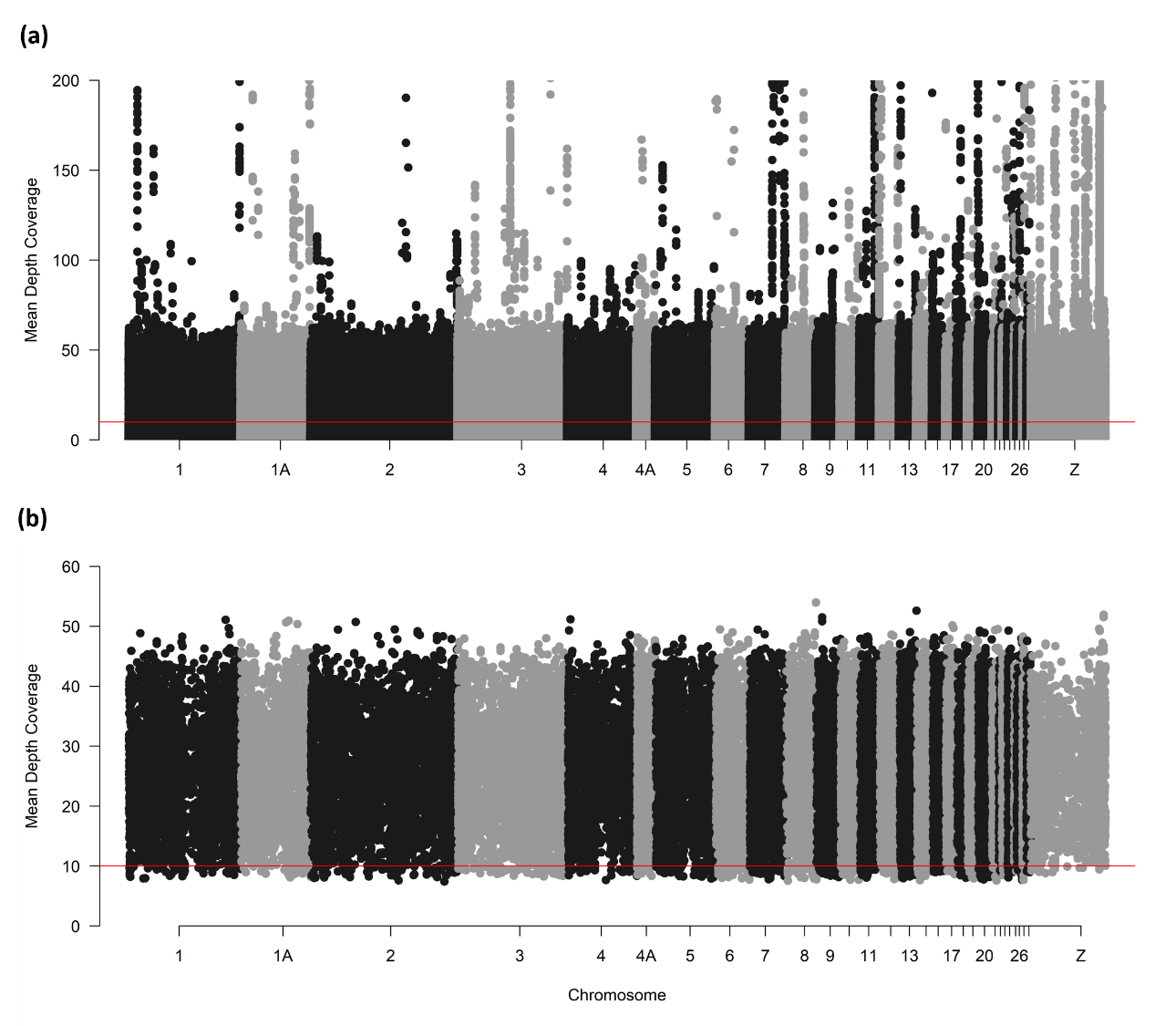


**Figure S1.** Manhattan plot showing the mean depth coverage per site across all SNPs. a) Mean depth coverage before filtering (mean = 10.84). b) Mean depth coverage after filtering (mean = 26.25). Black and grey colours distinguish different chromosomes numbered according to the zebra finch reference genome.


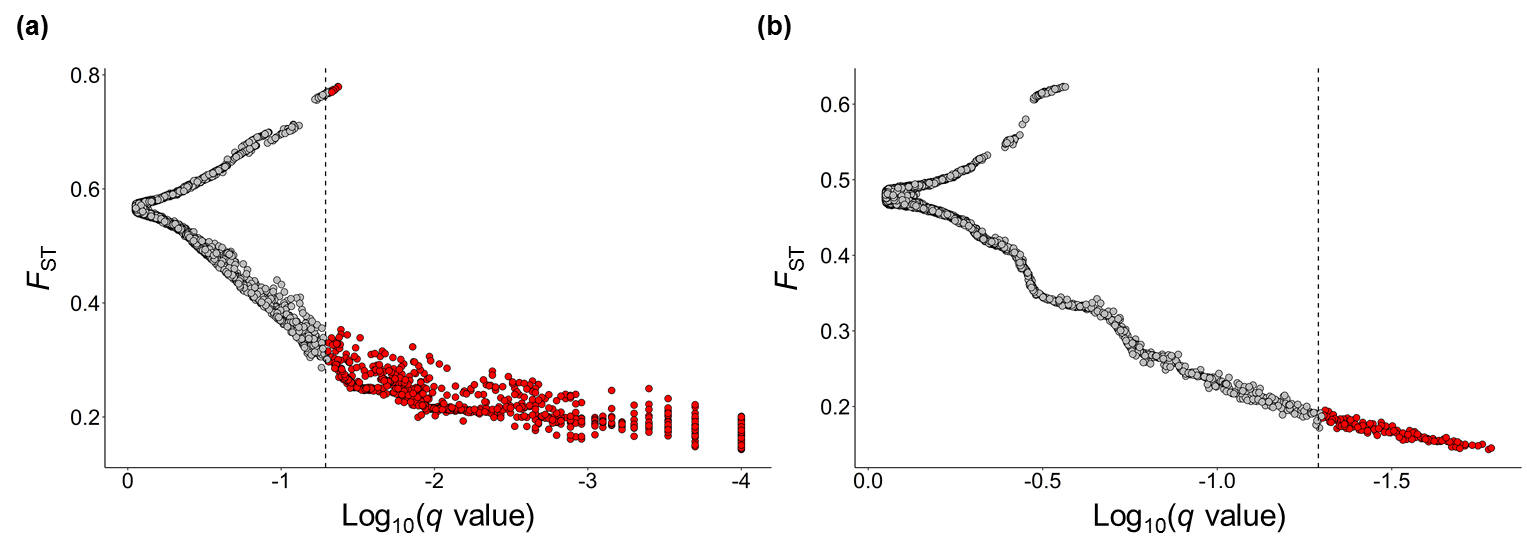


**Figure S2.** Identification of neutral SNPs and under selection using BayeScan v2.1. The horizontal axis indicates the log_10_ of the *q* value (FDR analog of the *p*-value) and the vertical axis indicates the mean *F*_ST_ between the different populations of chaffinch for each SNP used in this study. Each point represents a neutral SNP, and the points in red represent SNPs under selection. SNPs under selection with high *F*_ST_ values are under directional or disruptive selection, and SNPs under selection with low *F*_ST_ values are under stabilizing selection. (a) Neutral SNPs and under selection in all populations. (b) Neutral SNPs and under selection in the populations of Madeira and Canary Islands.


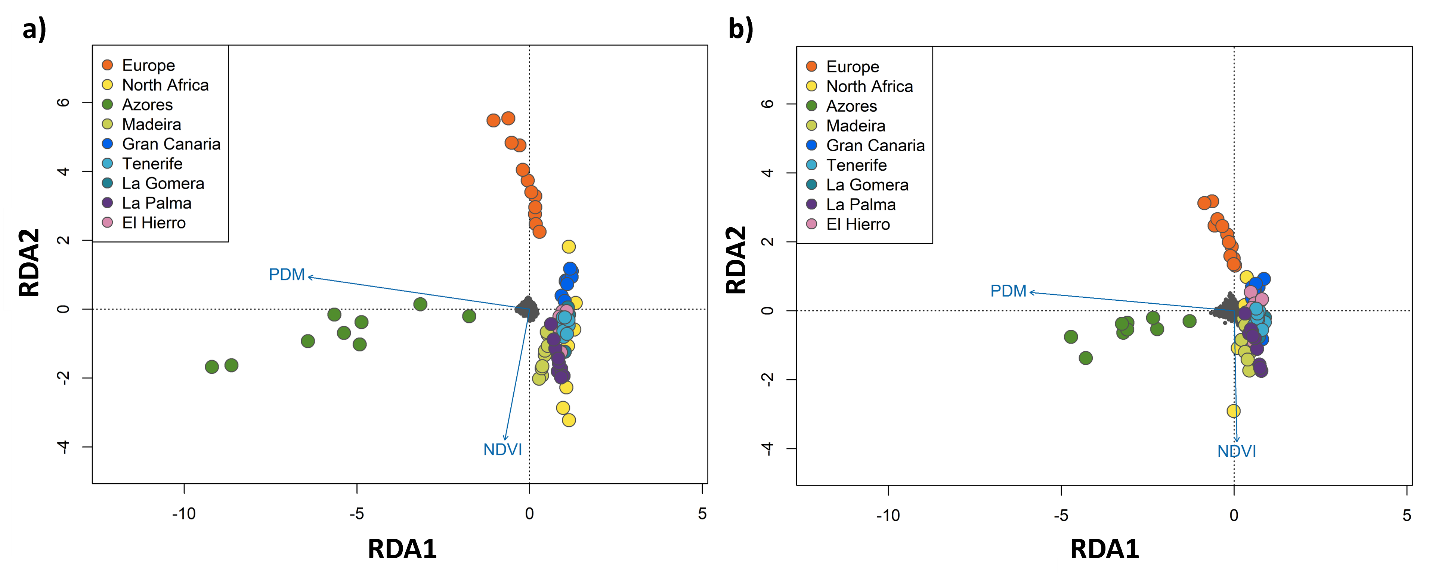


**Figure S3**. a) pRDA using all populations and SNPs with less than 20% missing data (31,144 SNPs) followed by imputation. b) pRDA using all populations and SNPs with less than 10% missing data (3,539 SNPs) followed by imputation.


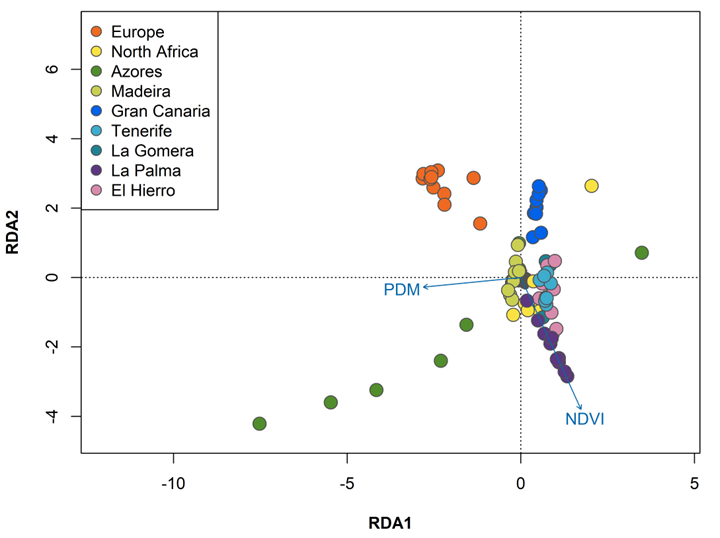


**Figure S4**. Partial RDA controlling for neutral genetic structure (using PCs 1 to 10) performed using the full dataset.

**References**

Borcard, D., Gillet, F., & Legendre, P. (2018). Numerical Ecology with R. Springer.

Chang, C. W., Fridman, E., Mascher, M., Himmelbach, A., & Schmid, K. (2022). Physical geography, isolation by distance and environmental variables shape genomic variation of wild barley (Hordeum vulgare L. ssp. spontaneum) in the Southern Levant. *Heredity*, 128(2), 107-119. <https://doi.org/10.1038/s41437-021-00494-x>

Cao, L. J., Gao, Y. F., Gong, Y. J., Chen, J. C., Chen, M., Hoffmann, A., & Wei, S. J. (2019). Population analysis reveals genetic structure of an invasive agricultural thrips pest related to invasion of greenhouses and suitable climatic space. *Evolutionary Applications*, 12(10), 1868-1880. <https://doi.org/10.1111/eva.12847>

Danecek, P., Auton, A., Abecasis, G., Albers, C. A., Banks, E., DePristo, M. A., ... & 1000 Genomes Project Analysis Group. (2011). The variant call format and VCFtools. *Bioinformatics*, 27(15), 2156-2158. [https://doi.org/10.1093/bioinforma tics/btr330](https://doi.org/10.1093/bioinforma%20tics/btr330)

Dormann, C. F., Elith, J., Bacher, S., Buchmann, C., Carl, G., Carré, G., ... & Lautenbach, S. (2013). Collinearity: a review of methods to deal with it and a simulation study evaluating their performance. *Ecography*, 36(1), 27-46. <https://doi.org/10.1111/j.1600-0587.2012.07348.x>

Dray, S., Bauman, D., Blanchet, G., Borcard, D., Clappe, S., Guénard, G., Jombart, T., Larocque, G., Legendre, P., Madi, N., & Wagner, H. H. (2022). _adespatial: Multivariate Multiscale Spatial Analysis_. R package version 0.3-20. – <<https://CRAN.R-project.org/package=adespatial>

Elshire, R. J., Glaubitz, J. C., Sun, Q., Poland, J. A., Kawamoto, K., Buckler, E. S., & Mitchell, S. E. (2011). A robust, simple genotyping-by-sequencing (GBS) approach for high diversity species. *PloS one*, 6(5), e19379. <https://doi.org/10.1371/journal.pone.0019379>

Forester, B. R., Lasky, J. R., Wagner, H. H., & Urban, D. L. (2018). Comparing methods for detecting multilocus adaptation with multivariate genotype–environment associations. *Molecular ecology*, 27(9), 2215-2233. <https://doi.org/10.1111/mec.14584>

Gibson, M. J., & Moyle, L. C. (2020). Regional differences in the abiotic environment contribute to genomic divergence within a wild tomato species. *Molecular Ecology*, 29(12), 2204-2217. <https://doi.org/10.1111/mec.15477>

Legendre, P., & Legendre, L. (1998). Numerical ecology: developments in environmental modelling. Developments in Environmental Modelling, 20(1). <https://doi.org/10.1017/CBO9781107415324.004>

McKenna, A., Hanna, M., Banks, E., Sivachenko, A., Cibulskis, K., Kernytsky, A., ... & DePristo, M. A. (2010). The Genome Analysis Toolkit: a MapReduce framework for analyzing next-generation DNA sequencing data. *Genome research*, 20(9), 1297-1303. <http://www.genome.org/cgi/doi/10.1101/gr.107524.110>

Oksanen, J., Simpson, G., Blanchet, F., Kindt, R., Legendre, P., Minchin, P., … & Weedon, J. (2022). _vegan: Community Ecology Package_. R package version 2.6-2. – <<https://CRAN.R-project.org/package=vegan>

Recuerda, M., Vizueta, J., Cuevas-Caballé, C., Blanco, G., Rozas, J., & Milá, B. (2021b). Chromosome-level genome assembly of the common chaffinch (Aves: *Fringilla coelebs*): a valuable resource for evolutionary biology. *Genome Biology and Evolution*, 13(4), evab034. <https://doi.org/10.1093/gbe/evab034>
